# EST-SSR Primer Development and Genetic Structure Analysis of *Psathyrostachys juncea* Nevski

**DOI:** 10.3389/fpls.2022.837787

**Published:** 2022-02-28

**Authors:** Zhen Li, Lan Yun, Zhiqi Gao, Tian Wang, Xiaomin Ren, Yan Zhao

**Affiliations:** ^1^College of Grassland, Resources and Environment, Inner Mongolia Agricultural University, Hohhot, China; ^2^Key Laboratory of Grassland Resources Ministry of Education, Hohhot, China

**Keywords:** *Psathyrostachys juncea* Nevski, forage grass, EST-SSR, genetic diversity, population structure

## Abstract

*Psathyrostachys juncea* is a perennial forage grass which plays an important role in soil and water conservation and ecological maintenance in cold and dry areas of temperate regions. In *P. juncea*, a variety of biotic and abiotic stress related genes have been used in crop improvement, indicating its agronomic, economic, forage, and breeding value. To date, there have been few studies on the genetic structure of *P. juncea*. Here, the genetic diversity and population structure of *P. juncea* were analyzed by EST-SSR molecular markers to evaluate the genetic differentiation related to tillering traits in *P. juncea* germplasm resources. The results showed that 400 simple sequence repeat (SSR) loci were detected in 2,020 differentially expressed tillering related genes. A total of 344 scored bands were amplified using 103 primer pairs, out of which 308 (89.53%) were polymorphic. The Nei’s gene diversity of 480 individuals was between 0.092 and 0.449, and the genetic similarity coefficient was between 0.5008 and 0.9111, with an average of 0.6618. Analysis of molecular variance analysis showed that 93% of the variance was due to differences within the population, and the remaining 7% was due to differences among populations. *Psathyrostachys juncea* materials were clustered into five groups based on population genetic structure, principal coordinate analysis and unweighted pair-group method with arithmetic means (UPGMA) analysis. The results were similar between clustering methods, but a few individual plants were distributed differently by the three models. The clustering results, gene diversity and genetic similarity coefficients showed that the overall genetic relationship of *P. juncea* individuals was relatively close. A Mantel test, UPGMA and structural analysis also showed a significant correlation between genetic relationship and geographical distribution. These results provide references for future breeding programs, genetic improvement and core germplasm collection of *P. juncea*.

## Introduction

**FIGURE 1:**
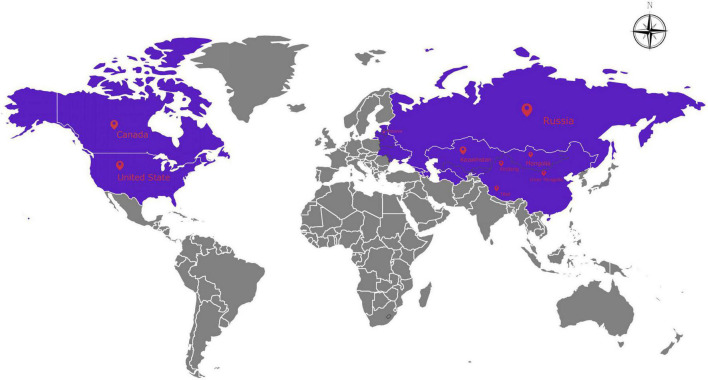
The distribution of 480 *Psathyrostachys juncea* accessions included in the present study.

*Psathyrostachys Nevski* is a genus of Gramineae grasses isolated from *Hordeum vulgare* L. by former Soviet botanists and contains about ten species. They are native to central and northern Asia’s inland areas and their distribution has spread to East Asia, Europe, and North America due to introduction and domestication (Yun et al., 2006; Figure 1). *Psathyrostachys* mainly grows in the north of the Tianshan Mountains in Xinjiang, but is also distributed in Inner Mongolia and Tibet in China. The *Psathyrostachys* genus is a rare diploid genus (2*n* = 2*x* = 14) in the Gramineae family, and it is a cross-pollinated, perennial bunch grass (Editorial Board of Flora of China, 1987), with clustered leaves, multiple tillers and a strong root system. *Psathyrostachys juncea* has a high feed value and is the only perennial forage species in this genus (Sbatella et al., 2011). *Psathyrostachys juncea* has been used in virus resistance research to improve wheat germplasm, including resistance to the yellow stunt virus (Wang et al., 2014; Ma et al., 2016; Hu et al., 2018) and resistance to stripe rust and take-all in wheat (Dong et al., 1992), and is also a precious genetic resource for improving abiotic stress resistance of crops and forage grass (Fu et al., 2003). It has extremely strong drought resistance and cold tolerance and can also restrain weeds, and can be established as an artificial grassland (Jefferson and Muri, 2007). Therefore, *P. juncea* is an excellent gramineous forage with agronomic, economic, forage, and breeding value (Liu et al., 2006).

Genetic diversity is an important component of biodiversity, and is the basis of ecosystem and species diversity (Bailey et al., 2009; Haddad et al., 2011; Zhang et al., 2015). Understanding the genetic diversity and structure of germplasm resources aids efficient and rational development, and the conservation and utilization of germplasm resources (Sork et al., 2013; Costa et al., 2016). Currently, researchers regard the study of perennial forage genetic diversity and population structure as crucial basic research, and such studies have been carried out on a variety of species. Genetic variation is frequently detected using morphological and agronomic characteristics, which often show multigenic inheritance strongly influenced by environmental factors. In addition to environmental differences, geographic isolation, phylogeographic patterns, gene flow and population dynamics also lead to selective pressures resulting in spatially structured genetic variation. Species will undergo adaptive evolution in phenotypes and phenology due to adaptive changes of genes in the genome (Feng et al., 2015; Rellstab et al., 2015). However, due to a lack of genomic information in a large number of non-model species, multiple candidate genes cannot be identified. These candidate genes play an important role in local adaptation in their genome, and thus cannot be ignored (Yong et al., 2017). Molecular marker analysis offers an efficient alternative to this approach (Kölliker et al., 2001). At present, the most common molecular markers for genetic diversity include SNP, RFLP, RAPD, ISSR, Simple Sequence Repeats (SSR), and AFLP (Li et al., 2021).

Simple sequence repeats molecular markers are co-dominant, which fits with the law of Mendel. It is easy to operate, has a high degree of reproducibility and reliability, and can reveal genetic differences between offspring and parents that are not affected by gene expression, cultivation conditions or environmental conditions (Wang et al., 2018; Daudi et al., 2021), and can show substantial polymorphism. SSR molecular markers are widely used in plant germplasm identification, genetic diversity, genetic linkage map construction, gene mapping and cloning, and in quantitative trait loci analysis (QTLs) (Ren et al., 2020; Chen et al., 2021; Jiang et al., 2021; Wu et al., 2021). Genetic diversity analysis using SSR molecular markers has been widely used in forage crops such as white clover (*Trifolium repens* L.), perennial ryegrass (*Lolium perenne* L.), and alfalfa (*Medicago sativa*) (Bolaric et al., 2005; Haddoudi et al., 2021; Wu et al., 2021; Zhang et al., 2021). In the *Psathyrostachys* genus, several researchers have reported analysis using SSR molecular markers on *Psathyrostachys huashanica*, an endangered and unique species with limited territory in the Huashan mountain area of China. Liu et al. (2014) analyzed the stripe rust resistance gene of *P*. *huashanica* using SSR molecular markers. The results showed that Yr H9020a might be a new gene for resistance to stripe rust, and the established molecular marker has potential to be used for molecular marker-assisted selection of the wheat stripe rust resistance gene. A large volume of literature shows that the results of SSR molecular markers can reveal the genetic relationship of closely related species and identify varieties (Wu et al., 2020).

The lack of DNA sequencing information for *P. juncea* hinders related breeding work. Previous study of *P. juncea* showed that genetic variation between individuals was much greater than the variation between populations (Xiong et al., 2020). This indicates that it is necessary to analyze and evaluate genetic differentiation on the individual level to identify desirable genes and breeding materials. As a typical bunch forage grass, previous study indicated that the variation coefficient of tiller number is the most significant among all agronomic traits, and there is a significant correlation between tiller number and aboveground biomass (Fan Y. K. et al., 2020). Expressed sequence tags are fundamental for the dissection of complex traits, and for estimating molecular diversity and population structure (Stavridou et al., 2021). As expressed sequence tag (EST)-SSR markers are derived from transcribed regions, they have high rates of successful amplification and associated gene annotations (Sun et al., 2021). SSR markers are cheap and easy to detect by polymerase chain reaction (PCR), so they are widely used in large population screening as valuable molecular markers. Therefore, this study aimed to develop EST sequence and EST-SSR markers for *P. juncea* related to the tiller number trait, and to analyze the relationship between genetic divergence and eco-geographic factors for this species. Furthermore, the genetic diversity and structure of the population were analyzed to characterize more basic molecular genetic information from *P. juncea* germplasm resources, in order to provide a scientific basis for cross-parent selection, and to construct the genetic map and analyze the genome-wide association of important agronomic traits of *P. juncea*. This research will provide references for the collection, protection and utilization of unique *P. juncea* germplasm resources.

## Materials and Methods

### Plant Materials

A total of 21 *P. juncea* accessions were selected as test materials, 19 accessions of which were provided by the U.S. National Plant Germplasm Resources Conservation System (NPGS), and two accessions by the China National Medium-term Gene Bank for Forage Germplasm. The variety 549118 ‘Mengnong No. 4’ was a variety selected from the cultivar ‘Bozoisky’ which was introduced from USDA-ARS-FRRL in 1984, and ‘Mengnong No. 4’ was registered by Inner Mongolia Agricultural University in 2009. The seeds of test materials were germinated in a greenhouse in January 2018 and transplanted to the field to establish the test plot in July 2018. The information of all accessions including eco-geographical conditions are shown in Supplementary Table 1. The test plot was located at the forage research station of Inner Mongolia Agricultural University, Hohhot, Inner Mongolia, China (111.41°E, 40.48°N). At least 30 plants in each accession were planted individually at spacings of 50 cm.

### DNA Extraction

A total of 480 individual plants were selected from 21 *P. juncea* accessions, ranged from 14 to 30 of each. The leaves were wiped with alcohol and deionized water and 1–2 fresh leaves per plant were cut into a 5 mL cryopreservation tube, and brought to the laboratory and stored at −80°C. DNA was extracted using a plant genomic DNA extraction kit (Tiangen Biotech Co., Ltd., Beijing, China). The quality and quantity of DNA was evaluated on 1% agarose gel with a NanoDropTM2000 Spectrophotometer (Thermo Fisher, Waltham, MA, United States). After all the measurements, the DNA stock solution was diluted to 50 ng/μL for SSR marker analysis and stored at −20°C.

### Expressed Sequence Tag Library Establishing and Differential Gene Expressions Simple Sequence Repeat Loci Identification

In order to establish EST library under the maximum genetic background, 17 accessions with significant difference in tiller number trait between individuals were selected from total 21 accessions. A group of thirty plants with dense tillers and another group of thirty plants with sparse tillers were selected from same 17 accessions (named sample 1 and sample 2, Supplementary Table 2). The normality tests of tiller related traits of sample one and sample two were listed in Supplementary Table 7 and simple statistics analysis were conducted using Origin 2019b (MicroCal, Northampton, MA, United States). 100 mg fresh tissues of the shoot meristem of each plant were briefly washed in deionized water and frozen in liquid nitrogen. Total RNA was extracted from tissue using the rapid plant RNA extraction kit and was treated with RNase-free DNase I at 37°C for 30 min to remove residual DNA (Takara, Otsu, Shiga, Japan). The RNA quality was verified using a 2100 Bioanalyzer (Agilent Technologies, Santa Clara, CA, United States) and confirmed using RNase-free agarose gel electrophoresis. The concentration of RNA was quantified with a Nano-drop 2000 (Thermo Fisher Scientific, Wilmington, DE, United States). Equal amounts of RNA of the thirty plants of each group were pooled to establish sample 1 and sample 2, and each sample had three replicates.

The cDNA library was constructed and sequenced by the Biomarker Biotechnology Corporation (Beijing, China). Briefly, the poly (A) mRNA was enriched via magnetic oligo (dT) beads and then broken into short fragments using an RNA Fragmentation Kit (Beckman Coulter, Brea, CA, United States). These cleaved mRNA fragments were used as templates for first strand cDNA synthesis using random hexamer primers. Second strand cDNA was synthesized with RNase H and DNA polymerase I. The library fragments were purified with an AMPure XP system (Beckman Coulter, Beverly, MA, United States). These short fragments were ligated to sequencing adapters, and the desired fragments were separated using AMPure XP beads. Next, the purified cDNA fragments were enriched via PCR. Finally, the cDNA libraries were sequenced using Illumina HiSeq™ 2000 and paired-end reads were generated. After obtaining high-quality sequencing data, the data was assembled to obtain unigenes.

According to the minimum length standard (unit size/minimum repetition time 1/12, 2/6, 3/5, 4/5, 5/5, and 6/5), unigenes over 1 Kb were analyzed using MISA^1^ to identify Simple Sequence Repeats. The primer pair for each SSR was designed according to differentially expressed genes and using Primer 3^2^ with the following criteria: primer lengths of 16–23 bases (optimum 20 bp), GC content of 40–70%, annealing temperature of 50–60°C, and PCR product size of 100–500 bp. The primers were synthesized by Sangon Biotech (Shanghai, China).

According to the relative expression level between the two samples, the differentially expressed genes were screened. A total of 2,020 significant differential gene expressions (DEGs) were identified, and 400 significant DEGs with SSR loci were screened. One SSR locus could synthesize three pairs of primers, and one pair of primers was selected for each locus. After removing 75 unigenes that were not suitable for designing primers, 325 pairs of primers were synthesized by Sangon Biotech (Shanghai, China). Eight individual plants from different populations were selected randomly, and used to test the amplification effect and polymorphism of 325 pairs of primers. A total of 103 primer pairs with polymorphism were selected and used in population genetic diversity and structure analysis (Supplementary Table 3).

### Polymerase Chain Reaction Amplification

A total of 103 selected SSR primer pairs were used in 480 individual plant DNA PCR reactions. The PCR reaction system used a 15 μL system for amplification, and reactions containing of 7.5 μL of 2 × Taq PCR MasterMix (including 3.0 mM MgCl_2_, 500 μM dNTPs, 0.1 U Taq DNA polymerase, 100 mM KCl, 20 mM Tris–HCl, Tiangen Biotech, Co., Ltd, Beijing, China), 1 μL of each forward and reverse primer, 50 ng of genomic DNA and 4.5 μL of ddH_2_O. PCR amplification was performed using an Applied Biosystems (Life Technologies Holdings Pte. Ltd, Singapore). The PCR conditions were as follows: pre-denaturation at 94°C for 4 min followed by 33 cycles of 94°C for 30 s, 51.5–58.5°C (depending on the Tm of the primer set used) for 30 s, and 72°C for 45 s, and finally extension at 72°C for 10 min. The fragment size of PCR amplified product was detected using a QIAxcel Advanced capillary electrophoresis instrument. The 100 bp to 2.5 kb DNA Size Marker and 15 bp to 3 kb Alignment Marker were selected as standard size markers (QIAGEN, Dusseldorf, Germany).

### Data Analysis

The BioCalculator software was used to accurately analyze a single data of amplified product by calculating the characteristics of various peaks, such as peak number, peak height, peak width and peak area. The DNA fragments amplified by the selected 103 pairs of primers were arranged in order from small to large, with high statistical resolution and clear bands, with “1” indicating presence of fragment or “0” indicating absence of fragment.

Taking band frequency less than 95%, the number of polymorphic bands (NPB) and the percentage of polymorphic bands (PPB) were calculated using Excel 2013. The GeneAlEx 6.503 (Peakall and Smouse, 2012) software was used to convert various file formats for different analysis and to calculate genetic diversity parameters, including the number of alleles (Na), the effective number of alleles (Ne), Shannon’s information index (I), Nei’s gene diversity (h), the number of private alleles, gene flow (Nm), and the F-Statistics (Fst). Genetic distance analysis, principal coordinate analysis (PCoA) and analysis of molecular variance (AMOVA) were conducted using GeneAlEx 6.503 software. AMOVA was carried out to partition the genetic variances into three levels: among populations, among individuals within populations, and within populations. The unweighted pair-group method with arithmetic means (UPGMA) cluster analysis was performed based on Nei’s unbiased genetic distance matrix with MEGA (Kumar et al., 2018). The Mantel test of the association between genetic distance and geographic distance were calculated by Geographic Distance Matrix Generator_v1.2.3 software (Ersts, 2010; Zhou et al., 2020). The geographical distance between different sites was calculated according to the latitude and longitude using the decimal degrees formula^3^ to detect whether the IBD (isolation by distance) patterns exist (Ersts, 2010).

The population genetic structure was analyzed using STRUCTURE 2.3.4 (Pritchard et al., 2000) software, using a model-based clustering algorithm that implements a Bayesian framework and the Markov Chain Monte Carlo (MCMC) algorithm. To confirm the optimum number of subpopulations (K), five independent runs for each value of K ranging from 2 to 10 were conducted (Wang M. L. et al., 2009). Each run consisted of a burn-in period of 10,000 steps followed by 100,000 MCMC iterations. The ΔK parameter, based on the rate of change in the log probability of data between successive K values, was estimated to determine the best K, based on the model developed by Evanno et al. (2005).

## Results

### Sequencing and *de novo* Assembly

**FIGURE 2:**
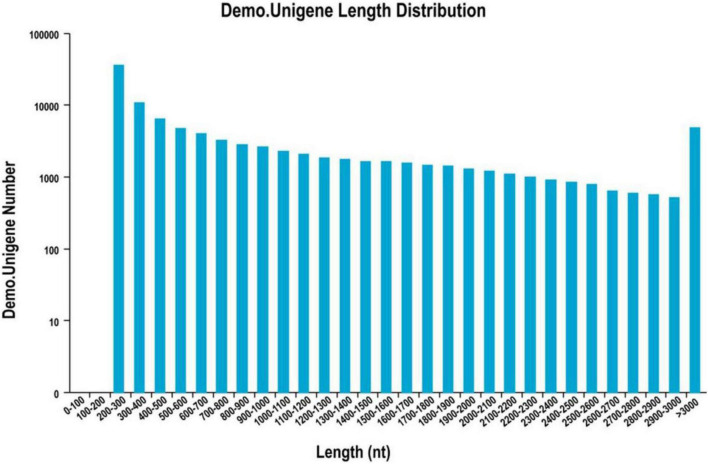
Unigene length distribution map.

The cDNA library was sequenced using the Illumina Hiseq high-throughput sequencing platform, and the transcriptome sequencing of six samples was completed. After removal of low-quality reads, a total of 38.81 GB of high-quality clean data was obtained. The total base number of clean data was 6,468,655,828, and the average Q30 and GC percentages were 92.37 and 54.02%, respectively. These clean reads were *de novo* assembled into 100,560 unigenes by the assembling program Trinity, and the total length of the unigenes was 91,562,881 bp, with an average length of 910 bp and the N50 of unigenes was 1,739 bp. Among all the unigenes, those with length ranging from 200 to 1,000 bp accounted for 70.28%, and 29.72% of the unigenes were more than 1,000 bp (Table 1). The length distributions of the unigenes are shown in Table 2 and Figure 2.

**TABLE 1:** Sequential assembly results.

Length range (bp)	All unigenes	Simple 1 unigenes	Simple 2 unigenes
200–300	36,038 (35.84%)	56,012 (41.20%)	54,734 (41.31%)
300–500	17,189 (17.09%)	33,725 (24.81%)	33,007 (24.91%)
500–1,000	17,444 (17.35%)	23,647 (17.39%)	23,082 (17.42%)
1,000–2,000	16,936 (16.84%)	14,270 (10.50%)	13,762 (10.39%)
2,000+	12,953 (12.88%)	8,294 (6.10%)	7,923 (5.98%)

**TABLE 2:** Summary of the analysis of *de novo* assembled EST-SSRs in *Psathyrostachys juncea*.

Category	Items	Number
Clean reads	Total clean reads (Gb)	38.81
	Total base number	6,468,655,828
	Q30 percentage (%)	92.37
	GC percentage (%)	54.02
Unigenes	Total number	100,560
	Total length (bp)	91,562,881
	Mean length (bp)	910.53
	N50 (bp)	1,739
EST-SSRs	Unigene length over 1 kb	29,889
	Number of SSR-containing sequences	9,300
	Significant DEG	2,020
	DEG with SSRs	400

### Frequency and Distribution of EST-SSRs

A total of 9,300 (31.12%) unigenes above 1 kb were identified as containing SSRs. EST-SSRs were of perfect repeat types, ranging from mononucleotide to hexanucleotide and among them, mononucleotide and trinucleotide repeats accounted for the largest proportion, at 40.30 and 36.11%, respectively and 400 EST-SSRs with compound repeats accounted for 4.3% of all sequences (Table 3).

**TABLE 3:** Density distribution of different types of simple sequence repeat (SSR).

Repeat type	Number
c	400
P1	3,748
P2	1,584
P3	3,358
P4	178
P5	21
P6	9
Total	9,300

*type: SSR type (p1, mono-nucleotide repeat SSR; p2, di-nucleotide repeat SSR; p3, tri-nucleotide repeat SSR; p4, tetra-nucleotide repeat SSR; p5, penta-nucleotide repeat SSR; p6, hexa-nucleotide repeat SSR; c, compound repeat SSR). Number, the number of identified SSR genes of this type.*

### The Polymorphism of Simple Sequence Repeat Markers

In the PCR reaction, a total of 344 loci were amplified using 103 SSR primer pairs among 480 individuals, of which 308 (89.53%) exhibited polymorphic patterns. The number of polymorphic loci for each primer combination varied from 1 to 6, with an average of 2.990 loci (Table 4). The amplified fragments of the 103 primer pairs were different in size and varied between 100 and 500 bp. All the primer pairs had a high Nei’s gene diversity value (h) and identified a high level of polymorphism. The percentage of polymorphic loci revealed different levels of polymorphisms ranging from 50 to 100%. The h value varied from 0.092 to 0.449 with an average of 0.274. The average effective Na per locus was 1.898. The primers also showed high Shannon’s information index (I), and the Shannon’s index of the 480 individual plant materials ranged from 0.173 to 0.640, with an average value of 0.420.

**TABLE 4:** Polymorphism analysis of 103 EST-SSR primers of *Psathyrostachys juncea*.

Primer	TNB	NPB	PPB	na	ne	*h*	*I*	Primer	TNB	NPB	PPB	na	ne	*h*	*I*
81	2	2	100.00%	1.833	1.409	0.254	0.395	70047	6	5	83.33%	1.900	1.645	0.360	0.526
516	4	3	75.00%	1.615	1.218	0.134	0.211	70343	2	2	100.00%	2.000	1.261	0.192	0.330
2249	2	2	100.00%	1.714	1.565	0.312	0.449	71974	4	4	100.00%	2.000	1.365	0.259	0.424
2947	2	2	100.00%	1.833	1.541	0.305	0.447	72525	4	3	75.00%	1.750	1.459	0.272	0.407
4435	4	3	75.00%	1.714	1.398	0.243	0.368	72655	6	6	100.00%	2.000	1.299	0.208	0.346
6022	4	3	75.00%	1.800	1.633	0.345	0.496	73069	4	4	100.00%	2.000	1.487	0.306	0.474
6181	2	2	100.00%	1.714	1.121	0.101	0.191	73311	2	2	100.00%	2.000	1.726	0.414	0.603
8649	6	5	83.33%	1.818	1.405	0.256	0.396	74564	2	2	100.00%	2.000	1.490	0.305	0.471
13109	4	4	100.00%	2.000	1.547	0.322	0.488	73848	4	4	100.00%	2.000	1.618	0.369	0.551
13558	6	3	50.00%	1.833	1.227	0.152	0.255	74428	4	4	100.00%	1.857	1.400	0.253	0.396
16533	4	3	75.00%	1.889	1.267	0.168	0.267	75415	4	4	100.00%	2.000	1.502	0.322	0.497
18679	4	3	75.00%	1.857	1.596	0.345	0.506	75587	2	2	100.00%	2.000	1.352	0.237	0.387
19368	4	3	75.00%	1.769	1.429	0.259	0.393	94279	2	2	100.00%	2.000	1.382	0.264	0.428
22073	2	2	100.00%	2.000	1.614	0.356	0.532	99009	2	2	100.00%	1.875	1.235	0.161	0.264
22271	4	4	100.00%	2.000	1.664	0.388	0.573	114416	2	2	100.00%	2.000	1.564	0.352	0.533
24537	4	4	100.00%	2.000	1.559	0.336	0.509	120572	4	4	100.00%	2.000	1.421	0.272	0.431
25029	4	3	75.00%	1.778	1.587	0.319	0.459	126480	2	2	100.00%	2.000	1.416	0.262	0.414
26249	2	2	100.00%	1.667	1.468	0.261	0.381	127259	2	2	100.00%	2.000	1.549	0.319	0.485
26620	6	6	100.00%	1.889	1.382	0.257	0.408	128934	4	3	75.00%	2.000	1.509	0.311	0.480
27806	2	2	100.00%	1.600	1.484	0.266	0.380	132067	4	4	100.00%	2.000	1.695	0.383	0.559
28161	2	2	100.00%	1.857	1.649	0.366	0.529	132162	4	3	75.00%	2.000	1.278	0.202	0.340
28324	4	4	100.00%	1.833	1.730	0.389	0.549	133160	2	1	50.00%	1.687	1.435	0.342	0.453
28410	2	2	100.00%	1.800	1.113	0.092	0.173	134436	4	4	100.00%	2.000	1.382	0.236	0.372
28553	2	2	100.00%	1.600	1.440	0.254	0.368	135127	2	2	100.00%	2.000	1.695	0.408	0.598
29223A	4	4	100.00%	1.625	1.484	0.272	0.392	137131	4	4	100.00%	2.000	1.466	0.293	0.458
29223B	4	4	100.00%	1.900	1.758	0.408	0.580	138171	4	4	100.00%	2.000	1.150	0.122	0.230
29697	4	4	100.00%	1.818	1.501	0.296	0.442	139768	4	3	75.00%	1.857	1.157	0.119	0.213
30766B	2	2	100.00%	2.000	1.404	0.248	0.388	139898	2	2	100.00%	2.000	1.650	0.367	0.542
35661	4	3	75.00%	1.750	1.136	0.105	0.189	139974	4	2	50.00%	2.000	1.673	0.398	0.586
37119	4	4	100.00%	1.800	1.656	0.358	0.511	140425	4	4	100.00%	2.000	1.751	0.405	0.583
37353	4	3	75.00%	1.583	1.158	0.114	0.191	141012	2	1	50.00%	2.000	1.544	0.341	0.521
38976	4	3	75.00%	1.917	1.242	0.139	0.221	141141	2	1	50.00%	1.750	1.227	0.169	0.287
40957	4	4	100.00%	1.923	1.469	0.275	0.420	142295	2	1	50.00%	2.000	1.605	0.342	0.512
43242	2	1	50.00%	1.833	1.465	0.277	0.420	142346	2	2	100.00%	2.000	1.302	0.214	0.355
43637	4	3	75.00%	2.000	1.427	0.284	0.450	142484	4	3	75.00%	1.818	1.207	0.144	0.244
44262	4	4	100.00%	1.900	1.489	0.291	0.442	142585	2	2	100.00%	2.000	1.654	0.383	0.566
45589	6	5	83.33%	1.750	1.455	0.274	0.412	142612	2	2	100.00%	2.000	1.392	0.272	0.438
45600	2	2	100.00%	2.000	1.307	0.208	0.348	143409	2	2	100.00%	2.000	1.599	0.351	0.526
46829	2	2	100.00%	2.000	1.722	0.403	0.589	144127	2	1	50.00%	2.000	1.614	0.348	0.519
51628	4	4	100.00%	1.875	1.240	0.178	0.305	144827	4	4	100.00%	2.000	1.634	0.375	0.559
58749	4	3	75.00%	1.900	1.430	0.270	0.419	144926	6	6	100.00%	2.000	1.394	0.254	0.408
60437	2	1	50.00%	1.667	1.364	0.219	0.334	146991	4	4	100.00%	2.000	1.765	0.430	0.620
61356	4	4	100.00%	2.000	1.540	0.336	0.513	149977	4	4	100.00%	2.000	1.543	0.346	0.528
63559	4	3	75.00%	2.000	1.590	0.357	0.535	163142	2	2	100.00%	2.000	1.827	0.449	0.640
64748	2	2	100.00%	1.857	1.387	0.259	0.408	163639	4	4	100.00%	2.000	1.827	0.443	0.633
65010	4	4	100.00%	1.857	1.283	0.204	0.339	165982	2	2	100.00%	2.000	1.522	0.324	0.498
65544	2	2	100.00%	2.000	1.364	0.252	0.411	167041	4	4	100.00%	2.000	1.485	0.304	0.473
66628	4	4	100.00%	2.000	1.472	0.312	0.489	185875	2	2	100.00%	2.000	1.611	0.361	0.541
66842	4	4	100.00%	2.000	1.532	0.339	0.520	193064	2	2	100.00%	2.000	1.707	0.403	0.589
67939	4	4	100.00%	2.000	1.470	0.274	0.417	194430	4	3	75.00%	1.857	1.321	0.210	0.339
68444	4	3	75.00%	1.917	1.587	0.340	0.503	194938	4	3	75.00%	1.714	1.410	0.229	0.341
69862	4	4	100.00%	2.000	1.378	0.263	0.427	Mean	3.340	2.990	89.53%	1.898	1.450	0.274	0.420

*TNB, total number of bands; NPB, number of polymorphic bands; PPB, percentage of polymorphic bands; Na, the number of alleles; Ne, the effective number of alleles; h, Nei’s gene diversity; I, Shannon’s Information index.*

### Genetic Similarity Analysis of *Psathyrostachys juncea* by Simple Sequence Repeat

Using the amplified markers of 480 individuals as the original data, genetic similarity coefficients were obtained (Supplementary Table 4). The genetic similarity coefficient varied from 0.5008 to 0.9111, with an average of 0.6618. The genetic similarity coefficient of accession 565052 from Russia and accession 598610 from Kazakhstan was the largest (0.9050), while that of accession 502576 from Russia and accession 531828 from the United States was the smallest (0.5008).

Analysis of the variation of the similarity coefficient of each plant in *P. juncea* material indicated that the genetic similarity coefficient between the accession 598610 individual plants from Kazakhstan had the greatest variation, which ranged from 0.5145 to 0.9111, indicating that this accession had great genetic differences and rich genetic diversity, while those plants of accession 531828 from the United States had the smallest variation, indicating that the genetic variation among individual plants of this accession had relatively small genetic differences and narrow genetic background (Table 5).

**TABLE 5:** Range of similarity coefficients on 480 simple plant of *Psathyrostachys juncea* germplasm.

Varieties code	Range of similarity coefficients	Mean	Varieties code	Range of similarity coefficients	Mean
PI 531828	0.6490–0.8878	0.7980	PI 565052	0.5236–0.9014	0.6653
PI 595135	0.6391–0.8924	0.7517	PI 531827	0.5217–0.9050	0.6146
PI 619487	0.5822–0.8829	0.6371	PI 272136	0.5190–0.8669	0.6182
CF 005043	0.5222–0.8829	0.7292	PI 502573	0.5316–0.9019	0.7166
PI 502577	0.5008–0.8636	0.7323	PI598614	0.5223–0.9036	0.6314
PI 549118	0.5031–0.8975	0.6581	PI 476299	0.5191–0.8904	0.6230
PI 502576	0.5009–0.8802	0.6458	PI 619565	0.5147–0.8919	0.6199
PI 565060	0.5104–0.8895	0.6072	PI 531826	0.5136–0.8981	0.6370
PI 565051	0.5138–0.8709	0.6343	PI 502572	0.5307–0.8959	0.6347
PI 578854	0.5390–0.8692	0.6471	PI 598610	0.5145–0.9111	0.6480
PI 619483	0.5272–0.8851	0.6482			

### Analysis of Genetic Diversity and Geographic Distribution of *Psathyrostachys juncea*

The 480 materials of *P*. *juncea* were divided into eight populations according to regions to compare the genetic diversity (Table 6). The Shannon’s information index and the change trend of genetic diversity followed the order: China > United States > Mongolia > Russia > Kazakhstan > Former Soviet Union > Estonia > Canada, the percentage of accessions with polymorphic locus from China was the largest (86.63%), and the smallest was from Canada (56.10%).

**TABLE 6:** Genetic diversity of *Psathyrostachys juncea* in different regions.

Populations	Accessions number	NPL	PPL (%)	na	ne	h	I
United States	42	278	80.81%	1.7932	1.4224	0.2517	0.3816
Canada	24	193	56.10%	1.5144	1.3003	0.1745	0.2615
China	98	298	86.63%	1.8534	1.4572	0.273	0.4142
Mongolia	72	295	85.73%	1.8298	1.4117	0.2487	0.3810
Kazakhstan	65	252	73.26%	1.7147	1.3711	0.2220	0.3369
Russia	107	281	81.69%	1.7919	1.3969	0.2405	0.3685
Former Soviet Union	45	247	71.80%	1.7042	1.3445	0.2092	0.3207
Estonia	27	238	69.19%	1.6531	1.3364	0.1993	0.3039

*NPL, number of polymorphic loci; PPL, percentage of polymorphic loci; Na, the number of alleles; Ne, the effective number of alleles; h, Nei’s gene diversity; I, Shannon’s information index.*

Analysis of molecular variance (AMOVA) was implemented to evaluate variance components among groups and individuals (Table 7), which showed highly significant differences (*P* < 0.05). Of the total accessions, 93% of the variance was due to differences within the accessions in the groups and the remaining 7% was due to differences among the groups. Of the individuals, 83% of the total genetic variance was due to differences within populations, while 5% was due to differences among populations and only 12% was ascribed to differences among individuals within the populations.

**TABLE 7:** Analysis of molecular variance (AMOVA) for 480 accessions of *Psathyrostachys juncea*.

	Source of variation	Degrees of freedom	Sum of squares	Mean square	Variance of components	Percentage variation (%)	F-statistic	*P*-value
Individuals	Among populations	7	2,861.437	408.777	3.295	5%	*F*_*st*_ = 0.142	<0.05
	Among individuals within populations	472	12,557.421	26.605	7.000	12%		<0.05
	Within populations	480	27,838.000	57.996	50.996	83%		<0.05
	Total	959	43,256.858		61.291	100%		
All accessions	Among populations	7	2,861.437	408.777	3.159	7%	*F*_*st*_ = 0.137	<0.001
	Within populations	952	40,395.421	42.432	42.432	93%		<0.001
	Total	959	43,256.858		45.591	100%		

**FIGURE 3:**
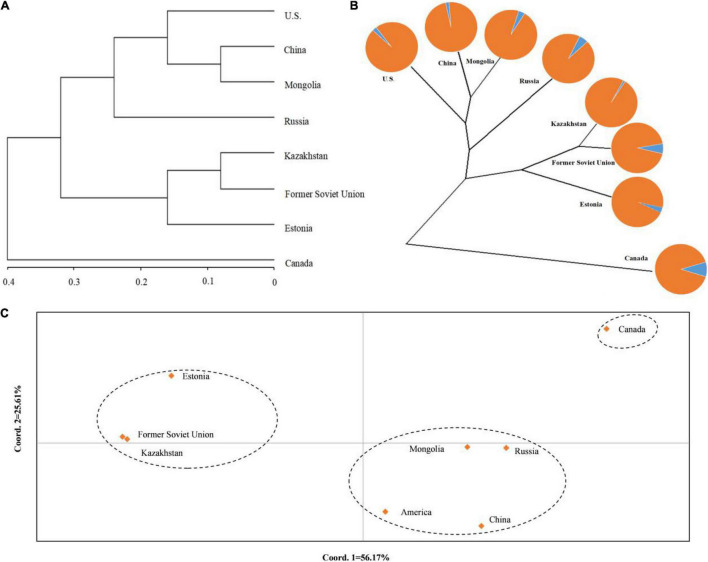
Population structure of eight regions populations of *Psathyrostachys juncea*. **(A)** The principal coordinates analysis (PCoA) of eight populations of *P. juncea*. **(B)** The unrooted tree based on Nei’s genetic distance for eight *P. juncea* populations. The pie chart reflects the distribution proportion of individuals of the regions populations in the two groups. **(C)** UPGMA analysis of eight populations of *P. juncea* based on Nei’s genetic distance.

To understand the genetic structure among the eight populations of *P. juncea*, PCoA and cluster analysis between the populations were carried out, based on the unweighted pair-group method with arithmetic means (UPGMA) on the regional populations (Figures 3A,C). In addition, an unrooted tree was drawn (Figure 3B). The results showed that the “Estonia,” “Kazakhstan,” and “Former Soviet Union” populations were clustered together; the “United States,” “Mongolia,” “Russia,” and “China” populations were clustered together; and “Canada” was one separate group.

The *N*_*m*_ and *F*_*st*_ among the eight regions were analyzed (Table 8). It was observed that the *N*_*m*_ between “Canada” and the others was relatively small and the *F*_*st*_ was large, which could explain why the “Canada” population was separately classified into one group.

**TABLE 8:** Gene flow (*N*_*m*_, above diagonal) and genetic differentiation coefficient (*F*_*st*_, below diagonal) between the eight regions.

	United States	China	Mongolia	Russia	Canada	Estonia	Kazakhstan	Former Soviet Union
United States	–	1.734	0.646	1.561	0.308	2.385	1.452	0.428
China	0.254	–	2.912	1.784	0.373	2.481	2.584	3.557
Mongolia	0.279	0.215	–	2.662	0.358	1.426	3.514	2.491
Russia	0.308	0.242	0.274	–	0.341	1.397	3.452	4.433
Canada	0.448	0.401	0.411	0.423	–	0.247	0.267	0.254
Estonia	0.394	0.342	0.370	0.386	0.503	–	2.364	1.346
Kazakhstan	0.356	0.300	0.327	0.356	0.483	0.407	–	2.422
Former Soviet Union	0.369	0.310	0.337	0.366	0.496	0.419	0.372	–

*F_st_, inter-population genetic fraction coefficient; N_m_, gene flow.*

**FIGURE 4:**
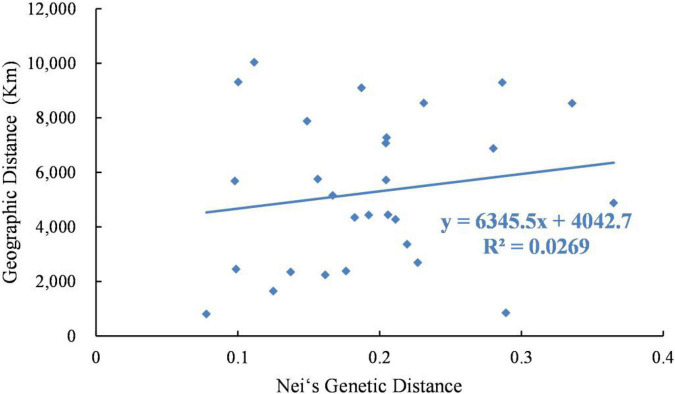
Relationship between genetic distance and geographic distance of 8 *Psathyrostachys juncea* populations.

Analysis using the Mantel test showed that there was a very significant relationship between genetic distance and geographical distance (*R*^2^ = 0.0269, *P* = 0.025). Therefore, the data showed that there was evidence of significant isolation by distance (Figure 4).

### Population Structure Analysis

**FIGURE 5:**
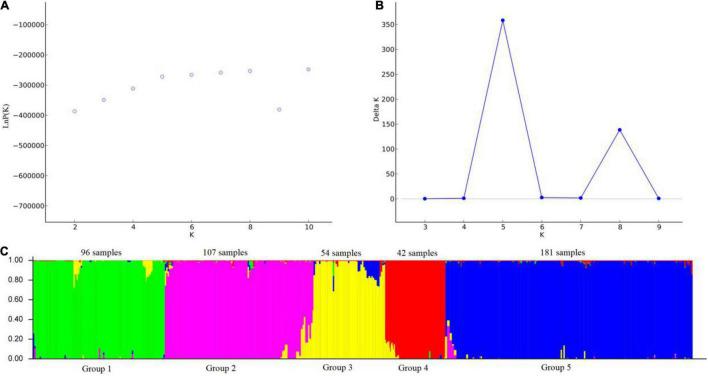
Analysis of the population structure of 480 *Psathyrostachys juncea* Individuals. **(A)** The average log-likelihood of *K*-value against the number of *K*; **(B)** Relations between the number of *K* and Δ*K*, based on the model developed by Evanno et al. (2005). **(C)** The population structure of *P. juncea* determined using STRUCTURE 2.3.4 software (Kölliker et al., 2001) (*K* = 5). Green area: Group 1; Purple area: Group 2; Yellow area: Group 3; Red area: Group 4; Blue area: Group 5.

The relationship among the accessions from the different groups and subgroups based on genetic distance was further explored by UPGMA cluster analysis, PCoA analysis, and genetic structure analysis. Markers of 103 SSR primer pairs scanned from 480 *P. juncea* individuals were analyzed using Structure 2.3.4 software. The line graphs were established using the abscissa as the *K* value (*K* = 2–10) and the ordinate as ln *P*(*K*). The ln *P*(*K*) increased constantly with increasing *K* and there was no maximum value, so the best number of subpopulations could not be determined (Figure 5A). The method of Evanno et al. (2005) was used to determine the best classification number. When the delta *K* value changed with the *K* value, an obvious peak can be identified as the best classification number. Structure cluster analysis showed that the ΔK value reached a peak when *K* = 5 (Figure 5B), which indicated that the 480 *P. juncea* individuals could be divided into five different groups.

*Psathyrostachys juncea* is completely cross-pollinated, so great differences in genetic variation exist within natural populations. In order to reveal the genetic components between individual plants as much as possible, the materials with *Q* value greater than 0.5 were classified into corresponding groups, which indicated that the genetic structure was relatively unitary. The materials with *Q* value less than 0.5 were considered complex (Supplementary Table 5). In group 1, 96 individual plants clustered into one category, and the accessions came from China and Mongolia in Asia. In group 2, 107 individuals were from Russia. In Group 3, 54 individuals were from Canada and Mongolia. In group 4, 42 individuals originated from the United States, and in group 5, 181 individuals were mainly concentrated in many countries in Asia and Europe, and were of multi-regional origin. Meanwhile, the genetic differentiation coefficient F_st_ was obtained to describe the heterozygosity level of the group alleles which is often used to measure the degree of population differentiation. The average *F*_*st*_ of the five groups followed the order group 4 (0.5487) > group 3 (0.4587) > group 2 (0.4442) > group 1 (0.4225) > group 5 (0.3370) (Supplementary Table 6), which showed that group 4 individuals have a high level of genetic differentiation and group 5 have the lowest level of genetic differentiation (Figure 5C).

**FIGURE 6:**
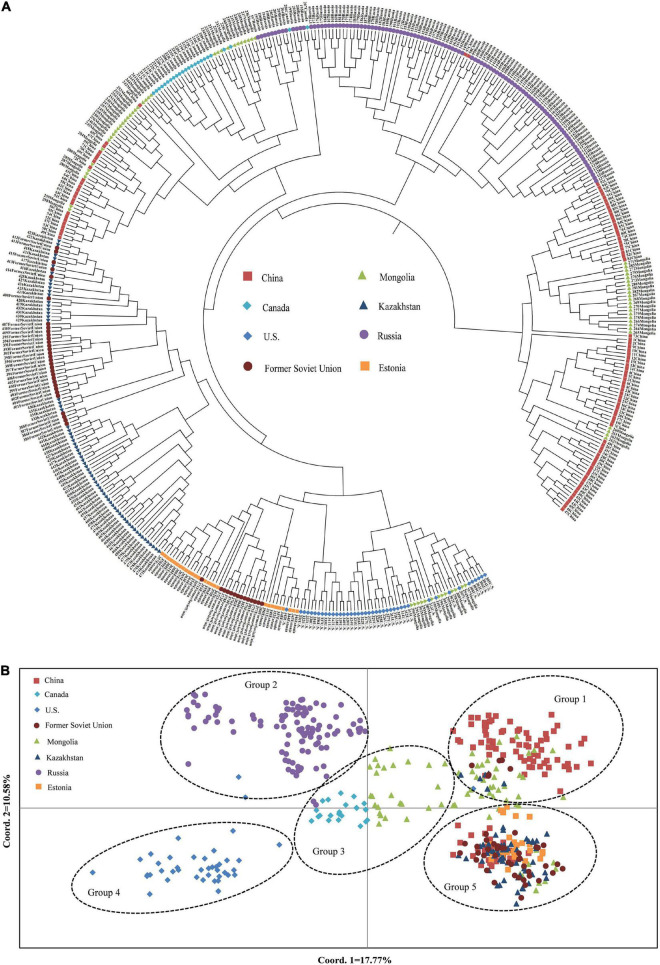
The UPGMA **(A)** and PCoA **(B)** analysis among 480 *Psathyrostachys juncea.*

The UPGMA dendrogram also showed that the accessions could be classified into five clusters (Figure 6A). The clustering of 480 *P. juncea* individual materials was basically in line with the results of population genetic structure analysis. Based on the UPGMA dendrogram, some individual plants from Russia were clustered with individual plants from Mongolia and Canada, while in the population structure analysis, plant materials from Russia were classified separately into group 2. The materials from Kazakhstan, Estonia, and the Former Soviet Union were clustered into one group in the UPGMA dendrogram, indicating that the genetic distance between materials from these three regions was small. In the cluster diagram, the materials from Russia were clustered into one group, and the materials from China and Mongolia were clustered into the same group. The classification of materials from Canada and the United States was extremely complex and they were clustered into different branches.

A total of 480 individual plants of *P. juncea* were analyzed based on the first and second principal components (Figure 6B). The distance of their positions in the figure represents the distance of genetic relationship. *Psathyrostachys juncea* individual plants can be classified into five groups, and the results of the PCoA were basically consistent with those of the UPGMA dendrogram and population structure analysis. The material distribution of individual plants in the five groups was relatively centralized, indicating that they were closely related to each other. In PCoA analysis, the materials from Mongolia were classified into multiple groups. The material sources of group 2 and group 4 had a relatively independent distribution, while groups 1, 3, and 5 had multi-distributed sources.

## Discussion

### The EST-SSR Loci Are Abundant in Tiller Related Differential Gene Expressions

Tillering is a major determinant of the forage yield of bunch grass, which is regulated by plant hormones, growth and development modes and environment. In recent years, new tillering genes and regulatory mechanisms of monocotyledons represented by Gramineae have been reported, which has deepened understanding of plant tillering. The regulation mechanism of plant tillering has been revealed from analysis of hormones, genes and transcription, which has provided a basis for the study of forage yield formation, environmental adaptation and the survival competitiveness of gramineous plants. Xu et al. (2020) reported that approximately 13,609 DEGs were identified from a high-tillering genotype and a low-tillering genotype of Orchard grass. In this research, *P. juncea* with dense tillers and sparse tillers were selected for transcriptome analysis. Among 100,560 unigenes, 9,300 genes with SSRs were identified. The significantly differentially expressed genes were screened according to the relative expression level between the two samples. A total of 2,020 significantly DEGs were identified, of which 400 were DEGs with SSR loci. In this study, we find abundant of EST-SSR exist in tiller trait related DEGs. These EST-SSR markers provide theoretical tools for the correlation analysis between tiller trait and molecular markers, and could play an important role in future breeding program to increase tiller number and forage yield. Furthermore, the *P. juncea* genome is more than 6.7 G base pair and limited genome sequence information was published (Li et al., 2020). SSR markers is easy to detect and analyze in a huge population with sufficient genetic variations. For genetic mapping and tiller trait QTL analysis, it is necessary to clarify the genetic distance of populations and individuals of different germplasms, especially to identify genetic differentiation between populations and individuals based on locus controlling tillering trait. It’s also necessary to analyze the genetic structure of populations to evaluate differentiation level of tiller trait related gene locus in *P. juncea*. The details of tillering trait transcriptome analysis of *P. juncea* are expected to be discussed in another manuscript.

### Molecular Markers Polymorphism Analysis

EST-SSR is the identification of SSR by electronic screening using existing EST sequences, followed by PCR detection (Zheng et al., 2020). Using EST to develop SSR avoids the cloning and sequencing steps in the process of developing SSR primers, makes full use of existing data, and reduces the development costs (Fan M. et al., 2020). EST-SSR is well conserved and has good versatility among different species, and can distinguish materials with relatively close genetic relationships (Huang and Lu, 2010). Liu et al. (2013) pointed out in the development and genetic diversity analysis of EST-SSR primers on panicle traits of wheat that the primers had high amplification efficiency and high polymorphism information content.

The polymorphism rate among the genotypes under exploratory conditions is considered a key factor in measurement of the diversity analysis efficiency of DNA markers (Athipat et al., 2019). A large number of studies have pointed out that the polymorphism of markers affects the level of genetic diversity of plants. Generally, the genetic parameters of tested materials were more reliable using primers with high polymorphism than those of primers with poor polymorphism. Zhang et al. (2019) analyzed the population and individuals of *P. juncea* germplasm using SSR primers distributed on seven linkage groups of barley, and reached the conclusion that SSR markers can provide a theoretical basis for genetic diversity research on *P. juncea*. However, in this study, for the first time EST-SSR markers were used to analyze the population structure of *P. juncea*, and 103 primer pairs were selected from 400 EST-SSR primers to mark the *P. juncea* population. The result showed that the polymorphism of EST-SSR markers developed from *P. juncea* was higher than SSRs from other related species, which indicates the population structure analysis was more accurate. The genetic diversity index in this research was slightly higher than that in Zhang et al. (2019). The high genetic diversity detected in this research may be due to the use of EST-SSR molecular markers developed for the genome of *P. juncea*, which can better distinguish gene loci. The Shannon information index (I) ranged from 0.173 to 0.640, with an average of 0.420 in our study, which was higher than that estimated by ISSRs for the related *P. huashanica* (*I* = 0.391). This finding indicates that the selected primers can objectively reveal the genetic diversity of *P. juncea* germplasm resources. Overall, the 103 EST-SSR primer pairs can provide an adequate analysis of the genetic differences among *P. juncea* materials.

### Genetic Variation of *Psathyrostachys juncea* Material and Genetic Diversity Among Subpopulations

Through simple statistics analysis of seven agronomic traits related to tillering of *P. juncea* in samples 1 and 2 group, the variation coefficient of nutritional tiller number was the most obvious in both samples (Supplementary Table 7), which suggested a relatively obvious genetic variation of tiller number within *P. juncea* population. It is consistent with the results of previous germplasm evaluation studies. Fan Y. K. et al.’s (2020) find tiller number trait has the most significant variation among all traits after evaluated 30 germplasm accession of *P. juncea* in field trail.

The genetic similarity coefficient (GS) is an index of the degree of similarity between individuals. When the genetic similarity coefficient is greater, the genetic relationship between materials is closer. The GS of 480 individual plants of *P. juncea* was analyzed, and the average value of GS was 0.6618, which showed that the genetic similarity level among individual plants of *P. juncea* was high. The materials with GS values ranging from 0.6 to 0.7 accounted for 60% of the total, indicating that the distribution of genetic diversity among materials was relatively centralized, the range of genetic variation was small, and the genetic relationship was close due to the limitation and centralization of the natural territory of *P. juncea*. Liu et al. (2009) analyzed the GS of 15 *P. juncea* materials using the ISSR molecular marker method and found that those with a GS range from 0.6 to 0.7 accounted for 37.4%. By comparing the average GS between wild materials and cultivated varieties, it was found that the GS value of cultivated varieties (0.667) was greater than that of wild materials (0.658), indicating that the genetic relationship between cultivated materials was closer and that of wild materials was more distant. The same detection was reported in the analysis of genetic similarity coefficient of wild materials and cultivated varieties of alfalfa (Chen and Shi, 2015). The genetic similarity coefficient of 88 *P. juncea* materials analyzed by RAPD molecular marker showed that the smallest genetic similarity coefficient was among Kazakhstan material and the greatest was among Russian material (Wei et al., 1997). That finding differs from the result in this study, indicating that in the process of genetic evolution, plant DNA has changed and produced a variety of rich germplasm banks with change in the surrounding environment. These differences also can be attributed to differences in molecular markers and plant materials used in each study.

In this research, based on the geographical origin of *P. juncea* accessions, accessions were clustered into eight subgroups for genetic structure analysis. The Shannon information index of each subpopulation ranged from 0.2615 to 0.4142, indicating a high genetic diversity level. The high genetic diversity in the *P. juncea* population may be related to the anemophily pollination of the species. Genetic variation within the populations (83–93%) was higher than that among populations (5–7%) in the present study. This result is consistent with the findings of previous studies of *P. juncea* based on AFLP analysis (77% within populations) (Xiong et al., 2020), Black cottonwood (*Populus deltoides*) based on SSR (84.88% within individuals) (Chen et al., 2020), white clover based on AFLP analysis (84% within cultivars) (Kölliker et al., 2001) and white clover based on SSR (86.5% within cultivars) (George et al., 2006). This is also consistent with the results of studies on other cross pollinating species, such as perennial ryegrass (Bolaric et al., 2005; Van Treuren et al., 2005). Our research showed that in the process of tillering, the genetic differences of *P. juncea* were mainly caused by individual variation.

Classically, a high degree of gene flow, which could neutralize interspecific differentiation and intraspecific genetic drift, is extremely common in cross-pollinated plants, thereby contributing to low genetic diversity among individuals or populations (Sui et al., 2009). In our study, the *P. juncea* gene flow (*N*_*m*_) between Canada and other countries was small, and the *N*_*m*_ between other countries was high. Ellstrand (1993) suggested that *N*_*m*_ < 1 indicates a high level of genetic differentiation among plant populations, while *N*_*m*_ > 1 indicates a low level of genetic differentiation among plant populations, and George et al. (2006) also found this result in cross pollinated white clover. Due to frequent gene exchange among subpopulations, the relationship between populations can be maintained, so the genetic differentiation between populations was not significant. In the process of evolution, *P. juncea* has undergone huge genetic variation and frequent exchange of genetic material with the materials of other populations. Therefore, germplasm resources with rich polymorphism and high genetic variation have been formed at the level of genetics and breeding. Germplasm collections and the introduction history of different countries may also affect gene flow and shape the new genetic structure of subpopulations.

The genetic structure of species is affected by the interaction of multiple factors, such as the transmission model of seeds and pollen, population demographic history, geological events, geographical or ecological barriers and divergent selection for environmental factors (Yang et al., 2017; Smith et al., 2020). Based on Nei’s GD, the eight populations were clustered into three groups. This clustering showed strong geographic regionality. China and Mongolia are also adjacent to Russia in geographical location and similar in climate environment, but the Canadian materials located in North America were clustered separately, which may be caused by the long-term accumulation of genetic variation in the process of adapting to the regional environmental conditions after the introduction of *P. juncea* germplasm from Eurasia to North America.

### Population Structure of *Psathyrostachys juncea* Germplasm Resources

In this study, according to the results of population genetic structure analysis, the materials with Q greater than 0.5 were assembled to five groups and the individual plants from the same region were also be clustered into different groups. In the UPGMA cluster diagram, some individual plants from Russia were clustered with individual plants from Mongolia and Canada, but in the population structure analysis Russian materials were clustered independently into group 2. The clustering may be the result of breeding and domestication, which has a great impact on the diversity structure. Selection and breeding tend to keep plants with economically valuable traits such as tillering ability. In addition, different environments can also cause genetic changes and thus affect the division of population structure. It may also be that the DNA of *P. juncea* materials contains hereditary substances of different individual plants, and the genetic information of all individuals was integrated by the population structure. UPGMA clustering was carried out based on the GD of *P. juncea* materials to cluster the closely related materials into one group, which may lead to the aggregation of materials with different genetic structure. Shen et al. (2010) analyzed the population genetic structure of 64 oat germplasms and also found that germplasm from the same region were clustered into different groups.

Through the analysis of population genetic structure of *P. juncea* individual materials from different countries and regions, it was found that the population structure of 480 *P. juncea* individual plants had a certain correlation with geographical distribution. The Mantel test of genetic distance and geographical distance among materials showed that there was a significant correlation (*R*^2^ = 0.0269, *P* = 0.025). This showed an IBD model over all sampling locations that was comparable with the results of Xiong et al.’s (2020) research, which analyzed the genetic diversity of eleven *P. juncea* wild germplasms using AFLP markers, and identified that the key factors inducing moderate genetic differentiation were isolation by distance (IBD). Generally, more opportunity for allelic exchange could be obtained by individuals of neighboring populations because gene flow will theoretically be obstructed by a longer geographical distance (Zhang et al., 2018). Our research showed that geographical isolation hindered gene exchange among individuals from different populations and promoted genetic differentiation. However, it was interesting that although Canada and the United States are geographically close, there were great differences in the genetic structure of these two subpopulations, and they were not clustered into the same groups in the group division in the UPGMA and PCoA analysis (Figure 6). The clusters show substantial overlap of different populations, except for those from Russia and the United States. Moreover, high values of the genetic mixture were also confirmed by structure analysis. This is mainly attributed to the cross pollination and self-incompatibility of plant species (Khan et al., 2009), human seed transplantation (Wang Z. Y. et al., 2009), different biological dispersal patterns and evolutionary forces (Chapman et al., 2010) and random dispersal in a region (Golkar and Mokhtari, 2018). In the long-term breeding process, gene exchange among germplasm resources is becoming more and more frequent, resulting in the decline of regional correlations and blurring of original geographical sources among materials, which reduces the genetic differentiation of *P. juncea* plants. Similar results have also been obtained for perennial ryegrass (Yu et al., 2011). In addition, compared to food crop species, *P. juncea* is generally used as a forage grass species, and has a relative short domestication and breeding history, and shows a certain level of consistency between genetic and geographical differentiation.

The *P. juncea* accessions in the present study were clustered into five groups based on PCoA, UPGMA, and STRUCTURE analyses. The classification of 480 *P. juncea* individual plant materials basically accorded with the analysis of population genetic structure, but there were some differences. This could be attributed to the different statistical principles applied in different methods (Lv et al., 2020; Wu et al., 2021). PCoA can provide a more valid classification based on the dissimilarity matrix of the original data, which is not strictly in line with the Hardy-Weinberg equilibrium assumption. Population structure analysis can better understand genetic diversity and estimate the variation of germplasm resources, which is conducive to their effective utilization. Structure assigns the accessions to subgroups probabilistically by a Bayesian clustering approach, and it is used for the subdivision of natural out-crossing populations. The clustering of accessions using UPGMA analysis was implemented based on genetic distance, which showed more detailed relationships among the accessions. Overall, these three methods could work together to provide a comprehensive understanding of the *P. juncea* population genetic structure. Moreover, the differences in *P. juncea* revealed can be assessed for their high breeding and hybridization advantages when selecting parent combinations, and provides one basis for the selection of *P. juncea* to make cross combinations with strong genetic differences.

## Conclusion

EST-SSR markers were used to analyze the genetic structure of *P. juncea* in this research. The findings of the study confirmed that *P. juncea* accessions had sufficient genetic diversity. The classification results, gene diversity and genetic similarity coefficients showed that the overall genetic relationship of *P. juncea* individuals was relatively close. Furthermore, the genetic relationship of *P. juncea* accessions had a significant correlation with geographical distribution. The result will provide molecular evidence for cross combination, marker assisted improvement, germplasm resources conservation and core germplasm collection for *P. juncea*.

## Data Availability Statement

The datasets presented in this study can be found in online repositories. The names of the repository/repositories and accession number(s) can be found below: National Center for Biotechnology Information (NCBI) BioProject database under accession number PRJNA789128.

## Author Contributions

ZL conducted the whole research and wrote the manuscript. ZG, TW, and XR helped in experiment and data analysis. LY designed the project, collected materials, and revised the manuscript. YZ helped to revised the manuscript. All authors contributed to the article and approved the submitted version.

## Conflict of Interest

The authors declare that the research was conducted in the absence of any commercial or financial relationships that could be construed as a potential conflict of interest.

## Publisher’s Note

All claims expressed in this article are solely those of the authors and do not necessarily represent those of their affiliated organizations, or those of the publisher, the editors and the reviewers. Any product that may be evaluated in this article, or claim that may be made by its manufacturer, is not guaranteed or endorsed by the publisher.

## Footnotes

1
https://webblast.ipk-gatersleben.de/misa/

2
http://primer3.ut.ee/

3
http://biodiversityinformatics.amnh.org/open_source/gdmg
