# Supplementary material for: EST-SSR Primer Development and Genetic Structure Analysis of Psathyrostachys juncea Nevski
Source: Front Plant Sci. 2022 Feb 28;13:837787. doi: 10.3389/fpls.2022.837787 (PMC8919075; doi:10.3389/fpls.2022.837787)
Supplement: Supplementary file 1 [file Table_1.DOCX]

# **Supplementary Table 1.** Geographic information of *P. juncea* populations

| **Accession** | **Varieties name** | **Individual plant number** | **Sample size** | **Longitude** | **Latitude** | **Origin** | **Germplasm preservation institutions** | **Cultivation** |
| --- | --- | --- | --- | --- | --- | --- | --- | --- |
| PI 549118 | ‘BOZOISKY SELECT’ (Mengnong No.4) | 1-23 | 23 | 111°46′0″E | 40°51′0″N | Utah, U.S. | National Medium term Gene Bank of Forage Germplasm, China | Cultivate |
| PI 595135 | X 93031 | 24-48 | 25 | 86°31′6″E | 43°36′43″N | Xin jiang,China | NPGS, U.S. | Wild |
| PI 531826 | D-3139 | 49-74 | 26 | 93°51′45″E | 42°22′6″N | China | NPGS, U.S. | Wild |
| CF 005043 | Shandan | 75-98 | 24 | 110°46′″E | 40°51′0″N | China | National Medium term Gene Bank of Forage Germplasm, China | Cultivate |
| PI 502577 | K 40175 | 99-118 | 20 | 113°51′0″E | 62°31′0″N | Russian Federation | NPGS, U.S. | Cultivate |
| PI 565052 | DJ-4155 | 119-137 | 19 | 112°50'28"E | 63°50'2"N | Russian Federation | NPGS, U.S. | Wild |
| PI 502576 | K 37772 | 138-165 | 28 | 113°51′0″E | 62°31′0″N | Russian Federation | NPGS, U.S. | Cultivate |
| PI 565060 | AJC-534 | 166-179 | 14 | 112°48'30"E | 63°21'2"N | Russian Federation | NPGS, U.S. | Wild |
| PI 565051 | DJ-4154 | 180-205 | 26 | 112°48'30"E | 63°21'2"N | Russian Federation | NPGS, U.S. | Wild |
| PI 578854 | SWIFT | 206-229 | 24 | 106°7′0″W | 52°2'0"N | Canada | NPGS, U.S. | Cultivate |
| PI 619483 | 96N-331 | 230-259 | 30 | 90°7′58″E | 49°29′13″N | Mongolia | NPGS, U.S. | Wild |
| PI 619487 | 96N-300 | 260-283 | 24 | 92°4′35″E | 49°51′50″N | Mongolia | NPGS, U.S. | Wild |
| PI 619565 | 96N-238 | 284-301 | 18 | 94°55′45″E | 49°22′50″N | Mongolia | NPGS, U.S. | Wild |
| PI 531828 | — | 302-326 | 25 | 113°51′45″W | 45°26′0″N | Idaho, U.S. | NPGS, U.S. | Wild |
| PI 476299 | VINALL | 327-343 | 17 | 99°59′55″W | 46°1′42″N | U.S. | NPGS, U.S. | Cultivate |
| PI 531827 | — | 344-370 | 27 | 24°28′0″E | 59°22′0″N | Estonia | NPGS, U.S. | Wild |
| PI 502573 | AR-163 | 371-393 | 23 | 164°9′5″E | 65°17′0″N | Former Soviet Union | NPGS, U.S. | Cultivate |
| PI 502572 | AR-142 | 394-415 | 22 | 164°9′5″E | 65°17′0″N | Former Soviet Union | NPGS, U.S. | Cultivate |
| PI 598614 | VIR U-0134923 | 416-434 | 19 | 50°30′0″E | 50°25′0″N | Kazakhstan | NPGS, U.S. | Wild |
| PI 272136 | — | 435-453 | 19 | 76°55′0″E | 43°19′0″N | Alma-Asa, Kazakhstan | NPGS, U.S. | Cultivate |
| PI 598610 | VIR U-0134973 | 454-480 | 27 | 58°45′0″E | 46°45′0″N | Kazakhstan | NPGS, U.S. | Wild |
