# Supplementary material for: EST-SSR Primer Development and Genetic Structure Analysis of Psathyrostachys juncea Nevski
Source: Front Plant Sci. 2022 Feb 28;13:837787. doi: 10.3389/fpls.2022.837787 (PMC8919075; doi:10.3389/fpls.2022.837787)
Supplement: Supplementary file 2 [file Table_2.DOCX]

# **Supplementary Table 2.** *Psathrostachys juncea* material used in the transcriptome

| **Order** | **Accession** | **Varieties name** | **Origin** | Number of plants used | |
| --- | --- | --- | --- | --- | --- |
|  |  |  |  | **Sample1** | **Sample2** |
| 1 | PI 549118 | ‘BOZOISKY SELECT’ (Mengnong No.4) | Utah, U.S. | 2 | 2 |
| 2 | PI 595135 | X 93031 | Xin jiang,China | 2 | 2 |
| 3 | PI 531826 | D-3139 | China | 1 | 1 |
| 4 | CF 005043 | Shandan | China | 2 | 2 |
| 5 | PI 502577 | K 40175 | Russian Federation | 2 | 2 |
| 6 | PI 565060 | AJC-534 | Russian Federation | 1 | 1 |
| 7 | PI 619487 | 96N-300 | Mongolia | 2 | 2 |
| 8 | PI 619565 | 96N-238 | Mongolia | 2 | 2 |
| 9 | PI 531828 | — | Idaho, U.S. | 2 | 2 |
| 10 | PI 531827 | — | Estonia | 2 | 2 |
| 11 | PI 502573 | AR-163 | Former Soviet Union | 2 | 2 |
| 12 | PI 272136 | — | Alma-Asa, Kazakhstan | 2 | 2 |
| 13 | PI 598610 | VIR U-0134973 | Kazakhstan | 1 | 1 |
| 14 | PI 499559 | D-2697 | China | 2 | 2 |
| 15 | PI 565044 | DJ-3890 | Russian Federation | 2 | 2 |
| 16 | PI 499560 | D-2562 | China | 1 | 1 |
| 17 | PI 665583 | KGZ-06-41-208 | Naryn, kyrgyzstan | 2 | 2 |
