# Supplementary material for: EST-SSR Primer Development and Genetic Structure Analysis of Psathyrostachys juncea Nevski
Source: Front Plant Sci. 2022 Feb 28;13:837787. doi: 10.3389/fpls.2022.837787 (PMC8919075; doi:10.3389/fpls.2022.837787)
Supplement: Supplementary file 3 [file Table_3.DOCX]

# **Supplementary Table 3.** Sequences of SSR primers

| **No.** | **Primer** | **Forward primer sequence(5' - 3')** | **Reverse primer sequence(5' - 3')** | **Tm/℃** |
| --- | --- | --- | --- | --- |
| 1 | 000081 | TCACTGCCTCTGCATCAATC | CCTCGGAGAAGCATGTGAAT | 55.0 |
| 2 | 000516 | CCAACGTCCATCCTTGAAGT | ACGGTTTGAACTGTTTTGCC | 54.5 |
| 3 | 002249 | GCGCACAGAGTTAATGGTGA | GTGAAGGGAAGAAGTGCCC | 56.0 |
| 4 | 002947A | ATCCTCCAACTTCCCAGTCC | AAGCAAGGGGAGAAGAGACC | 57.0 |
| 5 | 004435 | ATCTCGCCTCCTCTCTCCTC | GTTTCCACCTCCTCCTCCAC | 58.3 |
| 6 | 006022 | TTCCCAAACGGATCAAGTTC | AAAATTTGAGGCAAGCGTGA | 52.5 |
| 7 | 006181A | CCTTTTCCGTGCATACTGGT | CTGCGAGGGAATGATGGTAT | 55.0 |
| 8 | 008649 | GGCACTTATGACACCCCAAG | GGCGTGTGTATGCGAGTATG | 56.4 |
| 9 | 013109 | TCCTTCGGGATCCCTCTACT | ATGGTAGGGTTGGCTCTGTG | 57.0 |
| 10 | 013558 | ACTAGCGAAGCCCTGTCAAA | TCCCTAACTTCCTTCCCGTT | 56.0 |
| 11 | 016533C | TAGGTCAAGCCCAAGTTGCT | TCTTTACCGTCACCTCAGCC | 57.0 |
| 12 | 018679 | GCGACGGGAAGAAGAACTC | GGGTAGCCATGAGCACAAAG | 56.5 |
| 13 | 019368C | TGGATTGTTCTAGCTGCGTG | ACCTGTGTCTGTCCATTCCA | 55.5 |
| 14 | 022073A | CTGTCCCATCTCCCCTGTAA | CACTAGGAGGTCGGGCATAA | 56.4 |
| 15 | 022271 | GGTTCTTCCTTCCTTCCTCG | AGGGTAAGGGCCAGGTTTTA | 55.5 |
| 16 | 024537A | AACAGCCTCTGTTTGCTCGT | CCACCTCGTCCAGTCATTCT | 57.0 |
| 17 | 025029 | ACTGATATACCCGGTCGCTG | GGCCGGAGATTAGGCTTTAC | 56.5 |
| 18 | 026249 | CTGACCTGCATTCAGAGCCT | AGTACAGCATCCCGACCAAC | 57.5 |
| 19 | 026620 | CAACCTGTTGGGTTGGTAGG | CTCCCCAAGAAGCACGTATG | 56.0 |
| 20 | 027806 | GCAGTGTACATTTGCGCAGT | AACACTGACATGTGCAAGCC | 56.3 |
| 21 | 028161A | GCCCTACCTTAGCCCCTTT | GGCTCACTCACGATGGAGAT | 57.0 |
| 22 | 028324 | ATGAAGAGAATTTGCGGGTG | AATTTTGTAGGCGAGACGGA | 53.2 |
| 23 | 028410 | AAATGGAGAGGGGTTGTGTG | CACGCAGACATTGGAGTCAT | 55.0 |
| 24 | 028553 | TTGACAAATCAGTCAGGCCA | ACACCGAGAAATCCCATCAC | 54.5 |
| 25 | 029223A | GTCGTGATCCCTGGTCATGT | ACGACTTCTTCTCGCAGCAT | 57.0 |
| 26 | 029223B | TGGTACATGTGTCCACCACC | AAGAGCATCCTCGACAGCAC | 57.5 |
| 27 | 029697 | AGGCACAACTTGCTGCATAA | GCCACAACCCCATTAACAAC | 54.7 |
| 28 | 030766B | CTCTTGTGGCCCGGTATAAA | GGCATACCTGCTTGACGAAT | 55.0 |
| 29 | 035661 | TACGCACCATCACCACTTGT | CGACGACGACTACGAGTTCA | 56.6 |
| 30 | 037119 | CGCTTTATACTCCCGCAGTC | CATCGTAGGTGCACAACACC | 56.3 |
| 31 | 037353 | GAAGCTCTGGTTGTTGGAGG | AGCCTTCTTCTTCCTCCTGG | 56.5 |
| 32 | 038976 | GTTTAGGCGGGAGAGAAACC | CACCATCTCCCACAGCCTAT | 56.5 |
| 33 | 040957 | TGTTACGCCCCCAAAGTTAG | CGCCATAGCTTCAGTCAACA | 55.0 |
| 34 | 043242 | CGTGATGAAAACGCGTAAGA | GATGTTCATGGCAGCCTTTT | 53.2 |
| 35 | 043637B | GTGTGGCGATTCATGTTGAC | CAGAACGAAGATGTGCTCCA | 54.7 |
| 36 | 044262 | TACGACTTCCTCGAACACCC | GTCCAGTCGTCGATCTCCTC | 57.2 |
| 37 | 045589 | GACAGATTTGACTGGGAGCC | AAGGAACTCTTGAGTCCGCA | 56.0 |
| 38 | 045600A | TCTCGTAGACCGCCTTGAAT | GCCACCATAGCTTGCTTCTC | 56.0 |
| 39 | 046829B | GATCATGGGGAAGCGCAC | AACAACGACTCTCACCCACC | 57.2 |
| 40 | 051628 | CTTAAGTTGCATGTCCCCGT | TGGTCCATTCTCTTGGGAAG | 54.7 |
| 41 | 058749A | CCTCGTAGAAGACGTAGGCG | GACTTCAAGGCGCTGGTG | 57.5 |
| 42 | 060437 | GAAGAACAGGGACTGGACGA | TGGGGAAGAGTCTCACTTGG | 56.6 |
| 43 | 061356 | GTGTGCTACGCTTGGGTGT | CGTCCTCACTCTGTGGCTC | 58.5 |
| 44 | 063559 | ACATTTCCAGCCACATCCAC | AAGCCTTGTCCGTCTTGTTG | 55.6 |
| 45 | 064748 | GCCTGAAATCCAAGCACATT | CCCTGTTTAATCGGCTACCA | 54.0 |
| 46 | 065010 | GAACAGACGAAACCGCCC | TGGTGAGACTCTGCTCATGG | 57.0 |
| 47 | 065544 | GAGGGCGAGCCAAATAATC | ATGCTTCATTTGTTAGCGGG | 53.4 |
| 48 | 066628 | TGTGTTGGCCCGAACTAATA | TTCAATACAAGCCATTCAGGC | 53.4 |
| 49 | 066842 | AAGGTAGGGCCATTGTACCC | CTGGGTCATGAACGACTCCT | 57.0 |
| 50 | 067939 | GATCGGATTGTTGCTAGCCT | CCTTCCACTTCTTCTCGCAG | 55.4 |
| 51 | 068444 | CGAAGTCAAAAGCGAACACA | AATCATGGATCCCACCTGAA | 53.1 |
| 52 | 069862 | GGAGGTGGATCTACCGGG | GTGGGAGAAGACGACCATGT | 57.5 |
| 53 | 070047 | GTAAGCGGCAACCTCAGAAC | AACGTCTTATCCTCCTGCCC | 56.8 |
| 54 | 070343 | TTCTCTCACTGCACCCATTG | GCACCTTGCATTGCTGTCTA | 55.4 |
| 55 | 071974 | CTTGGCCTTGACGACCTTAG | GAAGTATGCCGACCTGAAGC | 56.0 |
| 56 | 072525 | ATCTTGCTCGAACGAACCAC | TGAAACCCCTTCTTCCTCCT | 55.5 |
| 57 | 072655A | TGTGGTACTACGCCTTGCTG | GACAGTAGAGCCAGCTTCCG | 58.0 |
| 58 | 073069 | AAACAAACTGTCCCTCACCG | CTTTCTGGCTCAGCACTTCC | 55.8 |
| 59 | 073311 | CACCCACCATAGCGAGTTG | GGGAAGTTGCTCTTCAGCAC | 56.5 |
| 60 | 073848A | TGGATACAATCCGTCTGCAA | GTATCACGGGCCAATTCATC | 53.5 |
| 61 | 074428A | TGGACAGCGATCTTTCACAG | GCCTCGTTGTCCAGATTGTT | 55.2 |
| 62 | 074564 | TCGGCTTCCTCTTCCTATCA | CAGGAGCTCCAAACCACTTT | 55.0 |
| 63 | 075415B | CCTCTCCACCCTGTCTGTTC | CTCTCTTCTTCGACCGTCCC | 57.7 |
| 64 | 075587A | GGTCAAGAATGGAGACTCGC | TCCTCTTCATTGAGGTCGCT | 55.8 |
| 65 | 094279 | AGACACGAGCAGTGCAGCTA | CTGCCCAGCTGTAAGCAGT | 58.5 |
| 66 | 099009 | TTGTCTGTTCTGGGGTCCTC | AGCAGAACCGATGACTTTGC | 56.5 |
| 67 | 114416 | GACGAGCCTGATATGTGCAA | CTTTATAGCCGTCCGGTGAC | 55.5 |
| 68 | 120572 | ACAAAGACGCCAATCCTGAC | CATCTGGATCCACTCGTCCT | 56.0 |
| 69 | 126480 | CACCACAGGAGATGCTGCTA | GGCGAGTCTGTTTTGGAGAC | 56.8 |
| 70 | 127259 | TCCTTCGGGATCCTACCTTT | GCTTGTGTTGATGCCTCTGA | 55.2 |
| 71 | 128934 | TTCGAAAGAGGAATTTTGCG | ACCAACCAAATCCAAATCCA | 51.1 |
| 72 | 132067 | TAAAAGAGGCATTGGTTGCC | ATGTTGCCGACATAGCATCA | 53.5 |
| 73 | 132162 | GTTGGCCACATTCCCTAAGA | TCAACGTTGCAGGTTACAGC | 55.3 |
| 74 | 133160 | AGTTCTTCAGCTCGACCCAA | GGTCCTTGAGGGTGAAGTTG | 56.0 |
| 75 | 134436 | TCTTTCTCTCCACACCCAGC | TGAGCTAGGACTCGCCAGAT | 57.5 |
| 76 | 135127 | CCGCAGTTGACATTCTCCTT | TATCCTTGGGGACTGGAACA | 55.2 |
| 77 | 137131 | GTTGTCGAGCCGGAACTTCT | TATCCACACTGTCGAGCTGC | 57.5 |
| 78 | 138171 | ATTCAACGCTGTAGCCCATC | GGTATTCTGCCTTTGGGTGA | 55.0 |
| 79 | 139768A | TCCGACATGTCATCCTTTGA | TTTCTGTCAACATCTGCATGG | 53.1 |
| 80 | 139898 | TCTCTGCTGTCTTCCCCTGT | GTCAAAGCAGAGAGCAGCCT | 58.0 |
| 81 | 139974 | GGTAGCAAAGTTTCCCATGC | AACAGCAATGAGCTCCGTTT | 54.7 |
| 82 | 140425 | GCATGCTATGGGCACAACTA | CTGGTCTGCACTATGCCAAA | 55.3 |
| 83 | 141012 | CTTCTTCTCCTCCTGATGCG | GGAGATCCTTCAAGTGCGAG | 55.6 |
| 84 | 141141 | AATTTCTTGGCGTGTTCCAC | CCAAATCGAATTGCTGTCCT | 52.8 |
| 85 | 142295B | GGAACGAGGAGGAGGAAGAC | GTATCTGAAGTCGGCGTCGT | 57.4 |
| 86 | 142346 | TTTCAGCTCGACCTCTTCGT | GAGATGGGTGAGGAGGAACA | 56.3 |
| 87 | 142484 | CCAACGTGTCTAGCTCCCTC | ACGTCACCCAGCTTGAGACT | 58.5 |
| 88 | 142585 | CTCCTGCTTTCTCGCTCG | GAACGTGTCCCAGATGGTCT | 56.8 |
| 89 | 142612A | GTTGCACAAGAACGCAAAGA | TCTTCATCCCCTTTGTCAGG | 54.0 |
| 90 | 143409 | GAAGAACCTTGCTGAGCGTC | GCTCGGTTATCTGCCTCTTG | 56.3 |
| 91 | 144127 | GAGCATGGTGGTGCAGATAA | GGATGCATGTTAGGGCTGTT | 55.3 |
| 92 | 144827 | AGTCTTCTTCCTCCCCTTGC | CATCTTCTGCAGCAGTCCCT | 57.4 |
| 93 | 144926B | TGGTGAGTTTACTCCCCCAC | ACCAGAATGCTGCTGCTCTT | 57.0 |
| 94 | 146991 | GGATGTAGGAGACCGCCAT | ACGAAGACGAAGGCTGGAT | 56.8 |
| 95 | 149977 | GATGATGACTGGATCACCCC | ACGCAGAGGAGGAAACTCAA | 55.3 |
| 96 | 163142 | TAGCAATTTGGGAAGGCATC | TCATTTTTGGAGCAGGGTTC | 52.8 |
| 97 | 163639 | GTATGGGGTGGCTTTCCTG | GTTGCTCCAACTCTTGAGCC | 56.3 |
| 98 | 165982 | ACGACCAGCACGTTCTTCTT | CCACTCATCACTCCCTCCTC | 57.0 |
| 99 | 167041 | GACCCTCAAGTTGCAATGGT | CCGCAAAGATGACACAGAGA | 55.2 |
| 100 | 185875 | GCCTGTAGTCTCGTCTTGGC | TACCTCTTCTGCTGCCACCT | 58.5 |
| 101 | 193064B | GCATCGATGACGACAGGAG | GATCTGGTGTCCTTCGGTGA | 56.3 |
| 102 | 194430 | GTCGTGGCGTCTACCCATAG | GAACGACGCACACTGGTTC | 57.7 |
| 103 | 194938 | GTTCCTCATTTGGGCGAGTA | GCACGGCATCTTTTAGCTTC | 54.6 |
