# Supplementary material for: EST-SSR Primer Development and Genetic Structure Analysis of Psathyrostachys juncea Nevski
Source: Front Plant Sci. 2022 Feb 28;13:837787. doi: 10.3389/fpls.2022.837787 (PMC8919075; doi:10.3389/fpls.2022.837787)
Supplement: Supplementary file 5 [file Table_5.DOCX]

# **Supplementary Table 5.** Q value of 480 *P. juncea* individuals in five groups

| **No.** | **Q1** | **Q2** | **Q3** | **Q4** | **Q5** | **Group** | **No.** | **Q1** | **Q2** | **Q3** | **Q4** | **Q5** | **Group** |
| --- | --- | --- | --- | --- | --- | --- | --- | --- | --- | --- | --- | --- | --- |
| 1 | 0.0009 | 0.9972 | 0.0011 | 0.0005 | 0.0004 | 1 | 241 | 0.0143 | 0.0004 | 0.0532 | 0.9317 | 0.0004 | 3 |
| 2 | 0.0009 | 0.9969 | 0.0007 | 0.0010 | 0.0005 | 1 | 242 | 0.0051 | 0.0003 | 0.0154 | 0.9788 | 0.0004 | 3 |
| 3 | 0.0007 | 0.9980 | 0.0003 | 0.0004 | 0.0006 | 1 | 243 | 0.0042 | 0.0008 | 0.1023 | 0.8922 | 0.0005 | 3 |
| 4 | 0.0014 | 0.9968 | 0.0004 | 0.0010 | 0.0005 | 1 | 244 | 0.1587 | 0.0013 | 0.1243 | 0.7144 | 0.0013 | 3 |
| 5 | 0.0004 | 0.9985 | 0.0004 | 0.0003 | 0.0004 | 1 | 245 | 0.1062 | 0.0008 | 0.1743 | 0.7176 | 0.0011 | 3 |
| 6 | 0.0011 | 0.9972 | 0.0004 | 0.0008 | 0.0004 | 1 | 246 | 0.0193 | 0.0025 | 0.1923 | 0.7851 | 0.0008 | 3 |
| 7 | 0.0005 | 0.9987 | 0.0003 | 0.0003 | 0.0002 | 1 | 247 | 0.0057 | 0.0147 | 0.1732 | 0.8057 | 0.0007 | 3 |
| 8 | 0.0006 | 0.9983 | 0.0004 | 0.0004 | 0.0004 | 1 | 248 | 0.1432 | 0.0011 | 0.1754 | 0.6794 | 0.0009 | 3 |
| 9 | 0.0004 | 0.9987 | 0.0003 | 0.0003 | 0.0002 | 1 | 249 | 0.0848 | 0.0090 | 0.1943 | 0.6904 | 0.0215 | 3 |
| 10 | 0.0007 | 0.9973 | 0.0007 | 0.0007 | 0.0005 | 1 | 250 | 0.0391 | 0.0013 | 0.1943 | 0.7646 | 0.0007 | 3 |
| 11 | 0.0008 | 0.9889 | 0.0031 | 0.0012 | 0.0060 | 1 | 251 | 0.0095 | 0.0004 | 0.1743 | 0.8154 | 0.0004 | 3 |
| 12 | 0.0164 | 0.9461 | 0.0006 | 0.0355 | 0.0013 | 1 | 252 | 0.0039 | 0.0900 | 0.2032 | 0.7016 | 0.0013 | 3 |
| 13 | 0.0006 | 0.9943 | 0.0004 | 0.0004 | 0.0043 | 1 | 253 | 0.0028 | 0.0015 | 0.2032 | 0.7919 | 0.0006 | 3 |
| 14 | 0.0006 | 0.9979 | 0.0005 | 0.0004 | 0.0006 | 1 | 254 | 0.0012 | 0.0004 | 0.2343 | 0.7637 | 0.0004 | 3 |
| 15 | 0.0006 | 0.9982 | 0.0002 | 0.0005 | 0.0004 | 1 | 255 | 0.0016 | 0.0002 | 0.2342 | 0.7638 | 0.0002 | 3 |
| 16 | 0.0006 | 0.9970 | 0.0006 | 0.0006 | 0.0013 | 1 | 256 | 0.0213 | 0.0014 | 0.0010 | 0.9757 | 0.0006 | 3 |
| 17 | 0.0010 | 0.9975 | 0.0004 | 0.0003 | 0.0007 | 1 | 257 | 0.0010 | 0.0003 | 0.0004 | 0.9981 | 0.0003 | 3 |
| 18 | 0.0004 | 0.9987 | 0.0002 | 0.0003 | 0.0003 | 1 | 258 | 0.0035 | 0.0002 | 0.0005 | 0.9952 | 0.0005 | 3 |
| 19 | 0.0004 | 0.9982 | 0.0004 | 0.0002 | 0.0009 | 1 | 259 | 0.0041 | 0.0005 | 0.0005 | 0.9945 | 0.0004 | 3 |
| 20 | 0.0013 | 0.9958 | 0.0009 | 0.0011 | 0.0008 | 1 | 260 | 0.0010 | 0.9967 | 0.0006 | 0.0004 | 0.0012 | 1 |
| 21 | 0.0004 | 0.9988 | 0.0002 | 0.0003 | 0.0003 | 1 | 261 | 0.0005 | 0.9965 | 0.0015 | 0.0008 | 0.0007 | 1 |
| 22 | 0.0003 | 0.9988 | 0.0002 | 0.0003 | 0.0003 | 1 | 262 | 0.0367 | 0.9578 | 0.0022 | 0.0019 | 0.0013 | 1 |
| 23 | 0.0008 | 0.9942 | 0.0009 | 0.0003 | 0.0038 | 1 | 263 | 0.0007 | 0.9964 | 0.0006 | 0.0005 | 0.0019 | 1 |
| 24 | 0.0023 | 0.8837 | 0.0014 | 0.0030 | 0.1097 | 1 | 264 | 0.0004 | 0.9983 | 0.0004 | 0.0003 | 0.0005 | 1 |
| 25 | 0.0034 | 0.9911 | 0.0005 | 0.0018 | 0.0032 | 1 | 265 | 0.0010 | 0.9975 | 0.0003 | 0.0008 | 0.0003 | 1 |
| 26 | 0.0009 | 0.9973 | 0.0003 | 0.0008 | 0.0006 | 1 | 266 | 0.0102 | 0.9869 | 0.0008 | 0.0008 | 0.0014 | 1 |
| 27 | 0.0040 | 0.9276 | 0.0197 | 0.0022 | 0.0465 | 1 | 267 | 0.0006 | 0.9968 | 0.0013 | 0.0007 | 0.0005 | 1 |
| 28 | 0.0015 | 0.9969 | 0.0005 | 0.0007 | 0.0005 | 1 | 268 | 0.0005 | 0.9980 | 0.0004 | 0.0007 | 0.0004 | 1 |
| 29 | 0.0020 | 0.9934 | 0.0017 | 0.0026 | 0.0004 | 1 | 269 | 0.0036 | 0.9900 | 0.0014 | 0.0033 | 0.0017 | 1 |
| 30 | 0.0020 | 0.6735 | 0.0028 | 0.3214 | 0.0003 | 1 | 270 | 0.0012 | 0.9964 | 0.0015 | 0.0005 | 0.0004 | 1 |
| 31 | 0.0029 | 0.8420 | 0.0014 | 0.1532 | 0.0005 | 1 | 271 | 0.0008 | 0.9902 | 0.0042 | 0.0005 | 0.0043 | 1 |
| 32 | 0.0009 | 0.8430 | 0.0013 | 0.1532 | 0.0016 | 1 | 272 | 0.0013 | 0.9970 | 0.0004 | 0.0006 | 0.0007 | 1 |
| 33 | 0.0013 | 0.7600 | 0.0028 | 0.2354 | 0.0005 | 1 | 273 | 0.0010 | 0.9977 | 0.0006 | 0.0004 | 0.0003 | 1 |
| 34 | 0.0010 | 0.8522 | 0.0006 | 0.1456 | 0.0006 | 1 | 274 | 0.0005 | 0.9982 | 0.0003 | 0.0005 | 0.0005 | 1 |
| 35 | 0.0053 | 0.8814 | 0.0008 | 0.1121 | 0.0004 | 1 | 275 | 0.0007 | 0.9981 | 0.0004 | 0.0005 | 0.0004 | 1 |
| 36 | 0.0012 | 0.8337 | 0.0007 | 0.1623 | 0.0021 | 1 | 276 | 0.0012 | 0.9976 | 0.0004 | 0.0005 | 0.0004 | 1 |
| 37 | 0.0025 | 0.9953 | 0.0005 | 0.0013 | 0.0003 | 1 | 277 | 0.0007 | 0.9981 | 0.0005 | 0.0005 | 0.0003 | 1 |
| 38 | 0.0005 | 0.9987 | 0.0003 | 0.0003 | 0.0002 | 1 | 278 | 0.0013 | 0.9976 | 0.0005 | 0.0004 | 0.0002 | 1 |
| 39 | 0.0256 | 0.9485 | 0.0019 | 0.0234 | 0.0006 | 1 | 279 | 0.0006 | 0.9983 | 0.0004 | 0.0003 | 0.0005 | 1 |
| 40 | 0.0006 | 0.9978 | 0.0003 | 0.0006 | 0.0007 | 1 | 280 | 0.0005 | 0.9985 | 0.0004 | 0.0003 | 0.0004 | 1 |
| 41 | 0.0007 | 0.9978 | 0.0006 | 0.0005 | 0.0004 | 1 | 281 | 0.0012 | 0.9904 | 0.0059 | 0.0003 | 0.0022 | 1 |
| 42 | 0.0008 | 0.9969 | 0.0009 | 0.0006 | 0.0008 | 1 | 282 | 0.0185 | 0.9383 | 0.0241 | 0.0013 | 0.0178 | 1 |
| 43 | 0.0014 | 0.9968 | 0.0004 | 0.0010 | 0.0005 | 1 | 283 | 0.0013 | 0.7435 | 0.0785 | 0.1489 | 0.0279 | 1 |
| 44 | 0.0016 | 0.9970 | 0.0004 | 0.0006 | 0.0004 | 1 | 284 | 0.0012 | 0.0046 | 0.9877 | 0.0061 | 0.0003 | 5 |
| 45 | 0.0009 | 0.9971 | 0.0006 | 0.0008 | 0.0005 | 1 | 285 | 0.0051 | 0.0006 | 0.9833 | 0.0043 | 0.0067 | 5 |
| 46 | 0.0007 | 0.9981 | 0.0004 | 0.0005 | 0.0003 | 1 | 286 | 0.0019 | 0.0004 | 0.9874 | 0.0096 | 0.0007 | 5 |
| 47 | 0.0020 | 0.9857 | 0.0020 | 0.0006 | 0.0097 | 1 | 287 | 0.0016 | 0.0007 | 0.9949 | 0.0022 | 0.0006 | 5 |
| 48 | 0.0012 | 0.9101 | 0.0321 | 0.0009 | 0.0558 | 1 | 288 | 0.0055 | 0.0006 | 0.9912 | 0.0019 | 0.0008 | 5 |
| 49 | 0.0410 | 0.0029 | 0.9537 | 0.0017 | 0.0006 | 5 | 289 | 0.0014 | 0.0010 | 0.9910 | 0.0063 | 0.0004 | 5 |
| 50 | 0.0012 | 0.0003 | 0.9971 | 0.0006 | 0.0007 | 5 | 290 | 0.0012 | 0.0004 | 0.9943 | 0.0027 | 0.0014 | 5 |
| 51 | 0.0023 | 0.0007 | 0.9950 | 0.0015 | 0.0006 | 5 | 291 | 0.0064 | 0.0007 | 0.9890 | 0.0035 | 0.0004 | 5 |
| 52 | 0.0184 | 0.0029 | 0.9520 | 0.0034 | 0.0233 | 5 | 292 | 0.0061 | 0.0012 | 0.9736 | 0.0187 | 0.0004 | 5 |
| 53 | 0.0031 | 0.0004 | 0.9939 | 0.0013 | 0.0013 | 5 | 293 | 0.0033 | 0.0004 | 0.9941 | 0.0018 | 0.0004 | 5 |
| 54 | 0.0023 | 0.0009 | 0.9947 | 0.0009 | 0.0012 | 5 | 294 | 0.0121 | 0.0007 | 0.9802 | 0.0064 | 0.0007 | 5 |
| 55 | 0.0061 | 0.0005 | 0.9888 | 0.0016 | 0.0030 | 5 | 295 | 0.0031 | 0.0122 | 0.9823 | 0.0017 | 0.0007 | 5 |
| 56 | 0.0021 | 0.0003 | 0.9953 | 0.0014 | 0.0009 | 5 | 296 | 0.0150 | 0.0007 | 0.9809 | 0.0012 | 0.0021 | 5 |
| 57 | 0.0009 | 0.0004 | 0.9954 | 0.0014 | 0.0019 | 5 | 297 | 0.0196 | 0.0022 | 0.9723 | 0.0040 | 0.0018 | 5 |
| 58 | 0.0016 | 0.0005 | 0.9963 | 0.0006 | 0.0009 | 5 | 298 | 0.0267 | 0.0019 | 0.8875 | 0.0825 | 0.0013 | 5 |
| 59 | 0.0010 | 0.0008 | 0.9961 | 0.0009 | 0.0012 | 5 | 299 | 0.0659 | 0.0098 | 0.9206 | 0.0028 | 0.0009 | 5 |
| 60 | 0.0032 | 0.0005 | 0.9934 | 0.0017 | 0.0012 | 5 | 300 | 0.0183 | 0.0059 | 0.9706 | 0.0044 | 0.0008 | 5 |
| 61 | 0.0014 | 0.0004 | 0.9961 | 0.0006 | 0.0015 | 5 | 301 | 0.0279 | 0.0017 | 0.9668 | 0.0013 | 0.0022 | 5 |
| 62 | 0.0016 | 0.0004 | 0.9965 | 0.0010 | 0.0005 | 5 | 302 | 0.7331 | 0.0033 | 0.2498 | 0.0103 | 0.0035 | 4 |
| 63 | 0.0011 | 0.0007 | 0.9956 | 0.0020 | 0.0007 | 5 | 303 | 0.8657 | 0.0020 | 0.0180 | 0.0028 | 0.1115 | 4 |
| 64 | 0.0008 | 0.0007 | 0.9971 | 0.0006 | 0.0008 | 5 | 304 | 0.9978 | 0.0007 | 0.0005 | 0.0006 | 0.0004 | 4 |
| 65 | 0.0013 | 0.0006 | 0.9959 | 0.0015 | 0.0006 | 5 | 305 | 0.9985 | 0.0005 | 0.0003 | 0.0003 | 0.0004 | 4 |
| 66 | 0.0025 | 0.0004 | 0.9946 | 0.0014 | 0.0011 | 5 | 306 | 0.9888 | 0.0018 | 0.0042 | 0.0019 | 0.0032 | 4 |
| 67 | 0.0019 | 0.0004 | 0.9934 | 0.0013 | 0.0030 | 5 | 307 | 0.9745 | 0.0011 | 0.0222 | 0.0004 | 0.0018 | 4 |
| 68 | 0.0017 | 0.0005 | 0.9955 | 0.0006 | 0.0017 | 5 | 308 | 0.9982 | 0.0005 | 0.0002 | 0.0006 | 0.0004 | 4 |
| 69 | 0.0016 | 0.0008 | 0.9950 | 0.0013 | 0.0013 | 5 | 309 | 0.9970 | 0.0012 | 0.0005 | 0.0010 | 0.0004 | 4 |
| 70 | 0.0008 | 0.0003 | 0.9976 | 0.0004 | 0.0009 | 5 | 310 | 0.9986 | 0.0006 | 0.0002 | 0.0003 | 0.0003 | 4 |
| 71 | 0.0010 | 0.0003 | 0.9970 | 0.0006 | 0.0011 | 5 | 311 | 0.9978 | 0.0008 | 0.0004 | 0.0005 | 0.0005 | 4 |
| 72 | 0.0017 | 0.0006 | 0.9960 | 0.0007 | 0.0010 | 5 | 312 | 0.9981 | 0.0005 | 0.0006 | 0.0004 | 0.0004 | 4 |
| 73 | 0.0009 | 0.0003 | 0.9976 | 0.0006 | 0.0005 | 5 | 313 | 0.9977 | 0.0006 | 0.0005 | 0.0004 | 0.0008 | 4 |
| 74 | 0.0299 | 0.0010 | 0.9556 | 0.0053 | 0.0083 | 5 | 314 | 0.9985 | 0.0005 | 0.0004 | 0.0004 | 0.0003 | 4 |
| 75 | 0.0026 | 0.8762 | 0.0577 | 0.0551 | 0.0084 | 1 | 315 | 0.9965 | 0.0014 | 0.0003 | 0.0014 | 0.0004 | 4 |
| 76 | 0.0006 | 0.9973 | 0.0011 | 0.0004 | 0.0005 | 1 | 316 | 0.9979 | 0.0005 | 0.0005 | 0.0006 | 0.0005 | 4 |
| 77 | 0.0012 | 0.9973 | 0.0002 | 0.0005 | 0.0008 | 1 | 317 | 0.9985 | 0.0004 | 0.0003 | 0.0004 | 0.0004 | 4 |
| 78 | 0.0044 | 0.9844 | 0.0056 | 0.0025 | 0.0031 | 1 | 318 | 0.9985 | 0.0006 | 0.0003 | 0.0003 | 0.0002 | 4 |
| 79 | 0.0026 | 0.9847 | 0.0014 | 0.0031 | 0.0082 | 1 | 319 | 0.9987 | 0.0004 | 0.0002 | 0.0004 | 0.0003 | 4 |
| 80 | 0.0006 | 0.9984 | 0.0004 | 0.0003 | 0.0004 | 1 | 320 | 0.9983 | 0.0005 | 0.0002 | 0.0007 | 0.0003 | 4 |
| 81 | 0.0013 | 0.9946 | 0.0027 | 0.0003 | 0.0011 | 1 | 321 | 0.9985 | 0.0005 | 0.0002 | 0.0004 | 0.0004 | 4 |
| 82 | 0.0006 | 0.9979 | 0.0004 | 0.0003 | 0.0007 | 1 | 322 | 0.9987 | 0.0004 | 0.0003 | 0.0003 | 0.0003 | 4 |
| 83 | 0.0004 | 0.9467 | 0.0003 | 0.0523 | 0.0003 | 1 | 323 | 0.9987 | 0.0004 | 0.0003 | 0.0004 | 0.0003 | 4 |
| 84 | 0.0005 | 0.9359 | 0.0006 | 0.0623 | 0.0007 | 1 | 324 | 0.9988 | 0.0003 | 0.0002 | 0.0003 | 0.0003 | 4 |
| 85 | 0.0006 | 0.7835 | 0.0003 | 0.2154 | 0.0002 | 1 | 325 | 0.9623 | 0.0014 | 0.0340 | 0.0010 | 0.0013 | 4 |
| 86 | 0.0007 | 0.8019 | 0.0003 | 0.1965 | 0.0006 | 1 | 326 | 0.9944 | 0.0006 | 0.0009 | 0.0008 | 0.0033 | 4 |
| 87 | 0.0005 | 0.8139 | 0.0004 | 0.1845 | 0.0007 | 1 | 327 | 0.9977 | 0.0008 | 0.0007 | 0.0004 | 0.0004 | 4 |
| 88 | 0.0007 | 0.8356 | 0.0006 | 0.1623 | 0.0008 | 1 | 328 | 0.9983 | 0.0004 | 0.0007 | 0.0003 | 0.0003 | 4 |
| 89 | 0.0016 | 0.8439 | 0.0003 | 0.1523 | 0.0019 | 1 | 329 | 0.9975 | 0.0005 | 0.0012 | 0.0005 | 0.0003 | 4 |
| 90 | 0.0015 | 0.8438 | 0.0004 | 0.1521 | 0.0022 | 1 | 330 | 0.9966 | 0.0011 | 0.0014 | 0.0006 | 0.0004 | 4 |
| 91 | 0.0586 | 0.7869 | 0.0007 | 0.1527 | 0.0011 | 1 | 331 | 0.9976 | 0.0003 | 0.0007 | 0.0011 | 0.0003 | 4 |
| 92 | 0.0004 | 0.8447 | 0.0003 | 0.1543 | 0.0003 | 1 | 332 | 0.9982 | 0.0004 | 0.0007 | 0.0004 | 0.0003 | 4 |
| 93 | 0.0004 | 0.9985 | 0.0004 | 0.0004 | 0.0003 | 1 | 333 | 0.9982 | 0.0003 | 0.0007 | 0.0005 | 0.0003 | 4 |
| 94 | 0.0004 | 0.9985 | 0.0005 | 0.0004 | 0.0002 | 1 | 334 | 0.9974 | 0.0005 | 0.0009 | 0.0007 | 0.0005 | 4 |
| 95 | 0.0010 | 0.9962 | 0.0004 | 0.0017 | 0.0006 | 1 | 335 | 0.9972 | 0.0006 | 0.0009 | 0.0008 | 0.0004 | 4 |
| 96 | 0.0015 | 0.9951 | 0.0005 | 0.0024 | 0.0005 | 1 | 336 | 0.9978 | 0.0004 | 0.0009 | 0.0005 | 0.0004 | 4 |
| 97 | 0.0006 | 0.9983 | 0.0005 | 0.0003 | 0.0003 | 1 | 337 | 0.9975 | 0.0005 | 0.0009 | 0.0006 | 0.0006 | 4 |
| 98 | 0.0005 | 0.9987 | 0.0002 | 0.0003 | 0.0003 | 1 | 338 | 0.9975 | 0.0006 | 0.0008 | 0.0004 | 0.0007 | 4 |
| 99 | 0.0777 | 0.0039 | 0.0318 | 0.0481 | 0.8384 | 2 | 339 | 0.9957 | 0.0006 | 0.0022 | 0.0010 | 0.0005 | 4 |
| 100 | 0.0215 | 0.0086 | 0.0104 | 0.0447 | 0.9149 | 2 | 340 | 0.9921 | 0.0010 | 0.0035 | 0.0029 | 0.0005 | 4 |
| 101 | 0.0176 | 0.0236 | 0.0026 | 0.0374 | 0.9188 | 2 | 341 | 0.8612 | 0.0012 | 0.0407 | 0.0964 | 0.0005 | 4 |
| 102 | 0.0153 | 0.0012 | 0.0394 | 0.0229 | 0.9211 | 2 | 342 | 0.9462 | 0.0007 | 0.0496 | 0.0030 | 0.0005 | 4 |
| 103 | 0.0005 | 0.0007 | 0.0006 | 0.0005 | 0.9978 | 2 | 343 | 0.8623 | 0.0011 | 0.0013 | 0.1338 | 0.0014 | 4 |
| 104 | 0.0005 | 0.0030 | 0.0003 | 0.0006 | 0.9955 | 2 | 344 | 0.0008 | 0.0004 | 0.9982 | 0.0004 | 0.0002 | 5 |
| 105 | 0.0006 | 0.0004 | 0.0004 | 0.0005 | 0.9982 | 2 | 345 | 0.0006 | 0.0007 | 0.9980 | 0.0005 | 0.0002 | 5 |
| 106 | 0.0019 | 0.0006 | 0.0018 | 0.0048 | 0.9909 | 2 | 346 | 0.0014 | 0.0007 | 0.9967 | 0.0009 | 0.0004 | 5 |
| 107 | 0.0009 | 0.0003 | 0.0014 | 0.0007 | 0.9968 | 2 | 347 | 0.0010 | 0.0003 | 0.9977 | 0.0008 | 0.0002 | 5 |
| 108 | 0.0022 | 0.0012 | 0.0065 | 0.0059 | 0.9843 | 2 | 348 | 0.0007 | 0.0003 | 0.9981 | 0.0007 | 0.0002 | 5 |
| 109 | 0.0012 | 0.0009 | 0.0003 | 0.0009 | 0.9967 | 2 | 349 | 0.0018 | 0.0004 | 0.9964 | 0.0011 | 0.0003 | 5 |
| 110 | 0.0005 | 0.0004 | 0.0005 | 0.0004 | 0.9982 | 2 | 350 | 0.0064 | 0.0033 | 0.9834 | 0.0053 | 0.0016 | 5 |
| 111 | 0.0005 | 0.0012 | 0.0019 | 0.0003 | 0.9961 | 2 | 351 | 0.0007 | 0.0007 | 0.9974 | 0.0005 | 0.0007 | 5 |
| 112 | 0.0005 | 0.0009 | 0.0004 | 0.0003 | 0.9978 | 2 | 352 | 0.0009 | 0.0013 | 0.9964 | 0.0010 | 0.0004 | 5 |
| 113 | 0.0008 | 0.0003 | 0.0007 | 0.0011 | 0.9971 | 2 | 353 | 0.0007 | 0.0004 | 0.9979 | 0.0007 | 0.0003 | 5 |
| 114 | 0.0015 | 0.0003 | 0.0020 | 0.0027 | 0.9936 | 2 | 354 | 0.0301 | 0.0044 | 0.9633 | 0.0018 | 0.0004 | 5 |
| 115 | 0.0010 | 0.0009 | 0.0006 | 0.0008 | 0.9967 | 2 | 355 | 0.0042 | 0.0073 | 0.9832 | 0.0048 | 0.0005 | 5 |
| 116 | 0.0021 | 0.0005 | 0.0006 | 0.0011 | 0.9958 | 2 | 356 | 0.0014 | 0.0244 | 0.9704 | 0.0021 | 0.0017 | 5 |
| 117 | 0.0008 | 0.0004 | 0.0007 | 0.0009 | 0.9972 | 2 | 357 | 0.0008 | 0.0006 | 0.9970 | 0.0007 | 0.0009 | 5 |
| 118 | 0.0014 | 0.0002 | 0.0010 | 0.0010 | 0.9964 | 2 | 358 | 0.0016 | 0.0025 | 0.9939 | 0.0015 | 0.0005 | 5 |
| 119 | 0.0007 | 0.0010 | 0.0006 | 0.0010 | 0.9967 | 2 | 359 | 0.0019 | 0.0007 | 0.9960 | 0.0011 | 0.0003 | 5 |
| 120 | 0.0025 | 0.0015 | 0.0010 | 0.0013 | 0.9937 | 2 | 360 | 0.0006 | 0.0154 | 0.9807 | 0.0004 | 0.0030 | 5 |
| 121 | 0.0014 | 0.0086 | 0.0005 | 0.0010 | 0.9884 | 2 | 361 | 0.0006 | 0.0003 | 0.9985 | 0.0004 | 0.0003 | 5 |
| 122 | 0.0027 | 0.0017 | 0.0589 | 0.1115 | 0.8253 | 2 | 362 | 0.0022 | 0.0076 | 0.9866 | 0.0029 | 0.0005 | 5 |
| 123 | 0.0030 | 0.0411 | 0.0595 | 0.0082 | 0.8882 | 2 | 363 | 0.0023 | 0.0009 | 0.9943 | 0.0020 | 0.0005 | 5 |
| 124 | 0.0023 | 0.0812 | 0.0029 | 0.0069 | 0.9068 | 2 | 364 | 0.0015 | 0.0006 | 0.9970 | 0.0006 | 0.0003 | 5 |
| 125 | 0.0019 | 0.0766 | 0.0112 | 0.0018 | 0.9085 | 2 | 365 | 0.0007 | 0.0009 | 0.9972 | 0.0007 | 0.0005 | 5 |
| 126 | 0.0521 | 0.0010 | 0.0062 | 0.0123 | 0.9285 | 2 | 366 | 0.0010 | 0.0313 | 0.9654 | 0.0018 | 0.0004 | 5 |
| 127 | 0.0024 | 0.0006 | 0.0007 | 0.0011 | 0.9952 | 2 | 367 | 0.0004 | 0.0011 | 0.9979 | 0.0003 | 0.0003 | 5 |
| 128 | 0.0030 | 0.0009 | 0.0029 | 0.0027 | 0.9905 | 2 | 368 | 0.0006 | 0.0013 | 0.9971 | 0.0006 | 0.0003 | 5 |
| 129 | 0.0012 | 0.0004 | 0.0005 | 0.0004 | 0.9975 | 2 | 369 | 0.0017 | 0.0011 | 0.9912 | 0.0057 | 0.0003 | 5 |
| 130 | 0.0005 | 0.0004 | 0.0007 | 0.0004 | 0.9979 | 2 | 370 | 0.0015 | 0.0005 | 0.9967 | 0.0011 | 0.0003 | 5 |
| 131 | 0.0011 | 0.0005 | 0.0004 | 0.0004 | 0.9977 | 2 | 371 | 0.0026 | 0.0006 | 0.9951 | 0.0010 | 0.0007 | 5 |
| 132 | 0.0009 | 0.0006 | 0.0006 | 0.0006 | 0.9972 | 2 | 372 | 0.0009 | 0.0026 | 0.9925 | 0.0024 | 0.0017 | 5 |
| 133 | 0.0004 | 0.0004 | 0.0003 | 0.0003 | 0.9986 | 2 | 373 | 0.0010 | 0.0005 | 0.9975 | 0.0007 | 0.0004 | 5 |
| 134 | 0.0016 | 0.0017 | 0.0268 | 0.0026 | 0.9673 | 2 | 374 | 0.0025 | 0.0008 | 0.9916 | 0.0046 | 0.0006 | 5 |
| 135 | 0.0013 | 0.0003 | 0.0008 | 0.0009 | 0.9967 | 2 | 375 | 0.0007 | 0.0009 | 0.9971 | 0.0008 | 0.0005 | 5 |
| 136 | 0.0006 | 0.0008 | 0.0003 | 0.0005 | 0.9978 | 2 | 376 | 0.0049 | 0.0008 | 0.9869 | 0.0068 | 0.0006 | 5 |
| 137 | 0.0028 | 0.0008 | 0.0003 | 0.0006 | 0.9955 | 2 | 377 | 0.0010 | 0.0009 | 0.9954 | 0.0016 | 0.0011 | 5 |
| 138 | 0.0006 | 0.0002 | 0.0003 | 0.0003 | 0.9987 | 2 | 378 | 0.0008 | 0.0006 | 0.9962 | 0.0013 | 0.0010 | 5 |
| 139 | 0.0004 | 0.0003 | 0.0002 | 0.0003 | 0.9989 | 2 | 379 | 0.0019 | 0.0018 | 0.9500 | 0.0413 | 0.0051 | 5 |
| 140 | 0.0008 | 0.0003 | 0.0022 | 0.0004 | 0.9964 | 2 | 380 | 0.0012 | 0.0004 | 0.9936 | 0.0039 | 0.0008 | 5 |
| 141 | 0.0008 | 0.0002 | 0.0006 | 0.0004 | 0.9979 | 2 | 381 | 0.0480 | 0.0036 | 0.9277 | 0.0152 | 0.0055 | 5 |
| 142 | 0.0004 | 0.0002 | 0.0003 | 0.0003 | 0.9988 | 2 | 382 | 0.0018 | 0.0013 | 0.9930 | 0.0018 | 0.0021 | 5 |
| 143 | 0.0007 | 0.0004 | 0.0003 | 0.0004 | 0.9982 | 2 | 383 | 0.0177 | 0.0030 | 0.9426 | 0.0117 | 0.0249 | 5 |
| 144 | 0.0007 | 0.0002 | 0.0003 | 0.0004 | 0.9983 | 2 | 384 | 0.0067 | 0.0004 | 0.9902 | 0.0021 | 0.0006 | 5 |
| 145 | 0.0009 | 0.0003 | 0.0003 | 0.0004 | 0.9980 | 2 | 385 | 0.0008 | 0.0007 | 0.9969 | 0.0010 | 0.0006 | 5 |
| 146 | 0.0039 | 0.0014 | 0.0005 | 0.0030 | 0.9912 | 2 | 386 | 0.0008 | 0.0004 | 0.9975 | 0.0007 | 0.0005 | 5 |
| 147 | 0.0009 | 0.0004 | 0.0005 | 0.0007 | 0.9975 | 2 | 387 | 0.0018 | 0.0021 | 0.9899 | 0.0036 | 0.0026 | 5 |
| 148 | 0.0038 | 0.0005 | 0.0003 | 0.0048 | 0.9906 | 2 | 388 | 0.0005 | 0.0007 | 0.9975 | 0.0007 | 0.0006 | 5 |
| 149 | 0.0179 | 0.0004 | 0.0005 | 0.0144 | 0.9667 | 2 | 389 | 0.0007 | 0.0002 | 0.9981 | 0.0003 | 0.0006 | 5 |
| 150 | 0.0010 | 0.0003 | 0.0003 | 0.0008 | 0.9977 | 2 | 390 | 0.0034 | 0.0007 | 0.9866 | 0.0016 | 0.0077 | 5 |
| 151 | 0.0009 | 0.0005 | 0.0005 | 0.0008 | 0.9973 | 2 | 391 | 0.0007 | 0.0003 | 0.9981 | 0.0004 | 0.0004 | 5 |
| 152 | 0.0008 | 0.0003 | 0.0003 | 0.0004 | 0.9983 | 2 | 392 | 0.0007 | 0.0004 | 0.9981 | 0.0003 | 0.0005 | 5 |
| 153 | 0.0007 | 0.0004 | 0.0002 | 0.0003 | 0.9984 | 2 | 393 | 0.0008 | 0.0003 | 0.9981 | 0.0004 | 0.0003 | 5 |
| 154 | 0.0006 | 0.0005 | 0.0004 | 0.0004 | 0.9981 | 2 | 394 | 0.0015 | 0.0005 | 0.9967 | 0.0007 | 0.0007 | 5 |
| 155 | 0.0021 | 0.0005 | 0.0020 | 0.0012 | 0.9943 | 2 | 395 | 0.0019 | 0.0010 | 0.9940 | 0.0020 | 0.0011 | 5 |
| 156 | 0.0004 | 0.0010 | 0.0003 | 0.0003 | 0.9980 | 2 | 396 | 0.0080 | 0.0041 | 0.9812 | 0.0017 | 0.0050 | 5 |
| 157 | 0.0007 | 0.0005 | 0.0003 | 0.0004 | 0.9981 | 2 | 397 | 0.0039 | 0.0009 | 0.9918 | 0.0010 | 0.0024 | 5 |
| 158 | 0.0008 | 0.0004 | 0.0005 | 0.0004 | 0.9979 | 2 | 398 | 0.0493 | 0.0009 | 0.9461 | 0.0008 | 0.0029 | 5 |
| 159 | 0.0013 | 0.0004 | 0.0005 | 0.0008 | 0.9970 | 2 | 399 | 0.0022 | 0.0004 | 0.9964 | 0.0006 | 0.0005 | 5 |
| 160 | 0.0014 | 0.0004 | 0.0057 | 0.0012 | 0.9913 | 2 | 400 | 0.0007 | 0.0003 | 0.9984 | 0.0003 | 0.0004 | 5 |
| 161 | 0.0006 | 0.0006 | 0.0003 | 0.0004 | 0.9980 | 2 | 401 | 0.0072 | 0.0006 | 0.9886 | 0.0011 | 0.0026 | 5 |
| 162 | 0.0008 | 0.0003 | 0.0004 | 0.0004 | 0.9981 | 2 | 402 | 0.0009 | 0.0003 | 0.9974 | 0.0005 | 0.0009 | 5 |
| 163 | 0.0008 | 0.0002 | 0.0005 | 0.0005 | 0.9979 | 2 | 403 | 0.0017 | 0.0004 | 0.9968 | 0.0006 | 0.0006 | 5 |
| 164 | 0.0030 | 0.0007 | 0.0009 | 0.0028 | 0.9925 | 2 | 404 | 0.0027 | 0.0004 | 0.9939 | 0.0008 | 0.0022 | 5 |
| 165 | 0.0019 | 0.0005 | 0.0007 | 0.0015 | 0.9953 | 2 | 405 | 0.0010 | 0.0010 | 0.9965 | 0.0004 | 0.0011 | 5 |
| 166 | 0.0059 | 0.0011 | 0.0049 | 0.0054 | 0.9828 | 2 | 406 | 0.0010 | 0.0006 | 0.9963 | 0.0008 | 0.0013 | 5 |
| 167 | 0.0034 | 0.0005 | 0.0026 | 0.0009 | 0.9926 | 2 | 407 | 0.0016 | 0.0005 | 0.9961 | 0.0007 | 0.0011 | 5 |
| 168 | 0.0008 | 0.0003 | 0.0005 | 0.0003 | 0.9981 | 2 | 408 | 0.0038 | 0.0005 | 0.9945 | 0.0007 | 0.0006 | 5 |
| 169 | 0.0029 | 0.0004 | 0.0011 | 0.0005 | 0.9951 | 2 | 409 | 0.0024 | 0.0006 | 0.9939 | 0.0007 | 0.0024 | 5 |
| 170 | 0.0012 | 0.0008 | 0.1460 | 0.0724 | 0.7796 | 2 | 410 | 0.0069 | 0.0014 | 0.9761 | 0.0009 | 0.0148 | 5 |
| 171 | 0.0008 | 0.0096 | 0.0193 | 0.0044 | 0.9659 | 2 | 411 | 0.0013 | 0.0005 | 0.9951 | 0.0005 | 0.0027 | 5 |
| 172 | 0.0011 | 0.0004 | 0.0008 | 0.0005 | 0.9972 | 2 | 412 | 0.0011 | 0.0009 | 0.9950 | 0.0004 | 0.0026 | 5 |
| 173 | 0.0008 | 0.0003 | 0.0012 | 0.0004 | 0.9972 | 2 | 413 | 0.0009 | 0.0003 | 0.9979 | 0.0003 | 0.0006 | 5 |
| 174 | 0.0010 | 0.0017 | 0.0292 | 0.0016 | 0.9665 | 2 | 414 | 0.0009 | 0.0006 | 0.9965 | 0.0003 | 0.0017 | 5 |
| 175 | 0.0040 | 0.0006 | 0.0012 | 0.0003 | 0.9940 | 2 | 415 | 0.0255 | 0.0029 | 0.9332 | 0.0084 | 0.0300 | 5 |
| 176 | 0.0011 | 0.0002 | 0.0007 | 0.0004 | 0.9976 | 2 | 416 | 0.0044 | 0.0003 | 0.9928 | 0.0010 | 0.0016 | 5 |
| 177 | 0.0009 | 0.0002 | 0.0003 | 0.0002 | 0.9985 | 2 | 417 | 0.0313 | 0.0026 | 0.9318 | 0.0020 | 0.0323 | 5 |
| 178 | 0.0018 | 0.0003 | 0.0003 | 0.0018 | 0.9958 | 2 | 418 | 0.0015 | 0.0006 | 0.9899 | 0.0006 | 0.0074 | 5 |
| 179 | 0.0111 | 0.0005 | 0.0006 | 0.0003 | 0.9875 | 2 | 419 | 0.0008 | 0.0005 | 0.9971 | 0.0009 | 0.0007 | 5 |
| 180 | 0.0039 | 0.0004 | 0.0010 | 0.2552 | 0.7395 | 2 | 420 | 0.0016 | 0.0006 | 0.9928 | 0.0009 | 0.0041 | 5 |
| 181 | 0.0003 | 0.0003 | 0.0002 | 0.0008 | 0.9983 | 2 | 421 | 0.0019 | 0.0005 | 0.9950 | 0.0014 | 0.0012 | 5 |
| 182 | 0.0003 | 0.0004 | 0.0004 | 0.0004 | 0.9986 | 2 | 422 | 0.0017 | 0.0004 | 0.9962 | 0.0012 | 0.0004 | 5 |
| 183 | 0.0002 | 0.0002 | 0.0002 | 0.0754 | 0.9240 | 2 | 423 | 0.0006 | 0.0002 | 0.9982 | 0.0008 | 0.0002 | 5 |
| 184 | 0.0002 | 0.0002 | 0.0003 | 0.1032 | 0.8961 | 2 | 424 | 0.0028 | 0.0009 | 0.9937 | 0.0017 | 0.0009 | 5 |
| 185 | 0.0002 | 0.0002 | 0.0006 | 0.1145 | 0.8845 | 2 | 425 | 0.0007 | 0.0003 | 0.9983 | 0.0004 | 0.0003 | 5 |
| 186 | 0.0002 | 0.0002 | 0.0002 | 0.1732 | 0.8262 | 2 | 426 | 0.0015 | 0.0009 | 0.9958 | 0.0011 | 0.0007 | 5 |
| 187 | 0.0002 | 0.0002 | 0.0003 | 0.1753 | 0.8240 | 2 | 427 | 0.0019 | 0.0012 | 0.9938 | 0.0012 | 0.0020 | 5 |
| 188 | 0.0003 | 0.0003 | 0.0003 | 0.0005 | 0.9987 | 2 | 428 | 0.0031 | 0.0006 | 0.9951 | 0.0009 | 0.0003 | 5 |
| 189 | 0.0002 | 0.0006 | 0.0002 | 0.0033 | 0.9956 | 2 | 429 | 0.0009 | 0.0004 | 0.9979 | 0.0005 | 0.0004 | 5 |
| 190 | 0.0031 | 0.0028 | 0.0003 | 0.0012 | 0.9926 | 2 | 430 | 0.0199 | 0.0013 | 0.9715 | 0.0070 | 0.0003 | 5 |
| 191 | 0.0002 | 0.0002 | 0.0003 | 0.0005 | 0.9988 | 2 | 431 | 0.0063 | 0.0015 | 0.9802 | 0.0112 | 0.0007 | 5 |
| 192 | 0.0002 | 0.0002 | 0.0002 | 0.0004 | 0.9991 | 2 | 432 | 0.0014 | 0.0009 | 0.9965 | 0.0008 | 0.0004 | 5 |
| 193 | 0.0002 | 0.0002 | 0.0003 | 0.0009 | 0.9983 | 2 | 433 | 0.0013 | 0.0021 | 0.9947 | 0.0016 | 0.0004 | 5 |
| 194 | 0.0002 | 0.0002 | 0.0002 | 0.0004 | 0.9990 | 2 | 434 | 0.0010 | 0.0004 | 0.9977 | 0.0006 | 0.0002 | 5 |
| 195 | 0.0003 | 0.0003 | 0.0002 | 0.0923 | 0.9069 | 2 | 435 | 0.0006 | 0.0013 | 0.7929 | 0.2035 | 0.0017 | 5 |
| 196 | 0.0002 | 0.0002 | 0.0002 | 0.0923 | 0.9071 | 2 | 436 | 0.0003 | 0.0030 | 0.5892 | 0.1043 | 0.3032 | 5 |
| 197 | 0.0002 | 0.0002 | 0.0003 | 0.0523 | 0.9470 | 2 | 437 | 0.0292 | 0.0003 | 0.7439 | 0.0532 | 0.1734 | 5 |
| 198 | 0.0002 | 0.0002 | 0.0011 | 0.3241 | 0.6744 | 2 | 438 | 0.0003 | 0.0002 | 0.8515 | 0.0156 | 0.1324 | 5 |
| 199 | 0.0006 | 0.0002 | 0.0006 | 0.3452 | 0.6534 | 2 | 439 | 0.0004 | 0.0005 | 0.9258 | 0.0010 | 0.0723 | 5 |
| 200 | 0.0004 | 0.0788 | 0.0007 | 0.4268 | 0.4933 | 2 | 440 | 0.0035 | 0.0005 | 0.9805 | 0.0152 | 0.0003 | 5 |
| 201 | 0.0005 | 0.0002 | 0.0008 | 0.1864 | 0.8121 | 2 | 441 | 0.0018 | 0.0237 | 0.9692 | 0.0024 | 0.0030 | 5 |
| 202 | 0.0002 | 0.0002 | 0.0005 | 0.3458 | 0.6533 | 2 | 442 | 0.0021 | 0.0011 | 0.9951 | 0.0010 | 0.0006 | 5 |
| 203 | 0.0005 | 0.0008 | 0.0003 | 0.2159 | 0.7825 | 2 | 443 | 0.0039 | 0.0028 | 0.9808 | 0.0116 | 0.0009 | 5 |
| 204 | 0.0002 | 0.0004 | 0.0002 | 0.3745 | 0.6247 | 2 | 444 | 0.0010 | 0.0075 | 0.9899 | 0.0008 | 0.0007 | 5 |
| 205 | 0.0002 | 0.0003 | 0.0006 | 0.3752 | 0.6237 | 2 | 445 | 0.0011 | 0.0019 | 0.9956 | 0.0009 | 0.0005 | 5 |
| 206 | 0.0017 | 0.0017 | 0.0005 | 0.5632 | 0.4329 | 3 | 446 | 0.0010 | 0.0009 | 0.9967 | 0.0010 | 0.0004 | 5 |
| 207 | 0.0009 | 0.0004 | 0.0002 | 0.9983 | 0.0002 | 3 | 447 | 0.0011 | 0.0004 | 0.9977 | 0.0006 | 0.0003 | 5 |
| 208 | 0.0010 | 0.0005 | 0.0003 | 0.9978 | 0.0004 | 3 | 448 | 0.0032 | 0.0008 | 0.9801 | 0.0051 | 0.0108 | 5 |
| 209 | 0.0013 | 0.0004 | 0.0005 | 0.9969 | 0.0010 | 3 | 449 | 0.0009 | 0.0005 | 0.9975 | 0.0005 | 0.0006 | 5 |
| 210 | 0.0161 | 0.1397 | 0.1535 | 0.6888 | 0.0019 | 3 | 450 | 0.0208 | 0.0053 | 0.9656 | 0.0038 | 0.0045 | 5 |
| 211 | 0.0012 | 0.0004 | 0.0003 | 0.9979 | 0.0003 | 3 | 451 | 0.0079 | 0.0018 | 0.9800 | 0.0083 | 0.0020 | 5 |
| 212 | 0.0300 | 0.0005 | 0.0007 | 0.9677 | 0.0012 | 3 | 452 | 0.0019 | 0.0003 | 0.9968 | 0.0006 | 0.0004 | 5 |
| 213 | 0.0617 | 0.0005 | 0.0004 | 0.9369 | 0.0004 | 3 | 453 | 0.0008 | 0.0007 | 0.9971 | 0.0005 | 0.0008 | 5 |
| 214 | 0.0016 | 0.0003 | 0.0003 | 0.9974 | 0.0003 | 3 | 454 | 0.0009 | 0.0005 | 0.9978 | 0.0004 | 0.0005 | 5 |
| 215 | 0.0009 | 0.0002 | 0.0002 | 0.9984 | 0.0002 | 3 | 455 | 0.0024 | 0.0004 | 0.9958 | 0.0006 | 0.0008 | 5 |
| 216 | 0.0009 | 0.0002 | 0.0002 | 0.9983 | 0.0003 | 3 | 456 | 0.0016 | 0.0004 | 0.9969 | 0.0005 | 0.0005 | 5 |
| 217 | 0.0014 | 0.0003 | 0.0002 | 0.9979 | 0.0002 | 3 | 457 | 0.0018 | 0.0004 | 0.9957 | 0.0008 | 0.0013 | 5 |
| 218 | 0.0559 | 0.0003 | 0.0003 | 0.9433 | 0.0003 | 3 | 458 | 0.0016 | 0.0005 | 0.9967 | 0.0005 | 0.0006 | 5 |
| 219 | 0.0011 | 0.0002 | 0.0002 | 0.9982 | 0.0002 | 3 | 459 | 0.0009 | 0.0004 | 0.9969 | 0.0003 | 0.0014 | 5 |
| 220 | 0.0368 | 0.0028 | 0.0003 | 0.9592 | 0.0009 | 3 | 460 | 0.0016 | 0.0004 | 0.9967 | 0.0006 | 0.0007 | 5 |
| 221 | 0.0257 | 0.0003 | 0.0004 | 0.9733 | 0.0004 | 3 | 461 | 0.0019 | 0.0003 | 0.9957 | 0.0007 | 0.0014 | 5 |
| 222 | 0.0023 | 0.0004 | 0.0005 | 0.9964 | 0.0004 | 3 | 462 | 0.0031 | 0.0004 | 0.9945 | 0.0013 | 0.0007 | 5 |
| 223 | 0.0054 | 0.0006 | 0.0005 | 0.9929 | 0.0006 | 3 | 463 | 0.0010 | 0.0003 | 0.9977 | 0.0006 | 0.0004 | 5 |
| 224 | 0.0016 | 0.0002 | 0.0002 | 0.9977 | 0.0003 | 3 | 464 | 0.0007 | 0.0003 | 0.9981 | 0.0004 | 0.0004 | 5 |
| 225 | 0.0017 | 0.0004 | 0.0002 | 0.9973 | 0.0004 | 3 | 465 | 0.0013 | 0.0003 | 0.9972 | 0.0008 | 0.0004 | 5 |
| 226 | 0.0027 | 0.0002 | 0.0003 | 0.9963 | 0.0005 | 3 | 466 | 0.0055 | 0.0010 | 0.9836 | 0.0020 | 0.0079 | 5 |
| 227 | 0.0016 | 0.0002 | 0.0003 | 0.9976 | 0.0003 | 3 | 467 | 0.0019 | 0.0003 | 0.9962 | 0.0011 | 0.0005 | 5 |
| 228 | 0.0520 | 0.0004 | 0.0005 | 0.9468 | 0.0003 | 3 | 468 | 0.0098 | 0.0018 | 0.9340 | 0.0042 | 0.0502 | 5 |
| 229 | 0.0031 | 0.0002 | 0.0003 | 0.9960 | 0.0004 | 3 | 469 | 0.0090 | 0.0074 | 0.9777 | 0.0029 | 0.0029 | 5 |
| 230 | 0.0045 | 0.0003 | 0.0002 | 0.9946 | 0.0004 | 3 | 470 | 0.0016 | 0.0004 | 0.9968 | 0.0007 | 0.0006 | 5 |
| 231 | 0.0047 | 0.0004 | 0.0003 | 0.9943 | 0.0003 | 3 | 471 | 0.0092 | 0.0010 | 0.9884 | 0.0010 | 0.0004 | 5 |
| 232 | 0.0020 | 0.0008 | 0.0005 | 0.9962 | 0.0006 | 3 | 472 | 0.0078 | 0.0004 | 0.9889 | 0.0021 | 0.0008 | 5 |
| 233 | 0.0015 | 0.0003 | 0.0004 | 0.9973 | 0.0004 | 3 | 473 | 0.0036 | 0.0006 | 0.9941 | 0.0012 | 0.0005 | 5 |
| 234 | 0.0026 | 0.0004 | 0.0002 | 0.9963 | 0.0005 | 3 | 474 | 0.0033 | 0.0010 | 0.9914 | 0.0017 | 0.0026 | 5 |
| 235 | 0.0036 | 0.0004 | 0.0007 | 0.9948 | 0.0005 | 3 | 475 | 0.0639 | 0.0014 | 0.9153 | 0.0130 | 0.0064 | 5 |
| 236 | 0.0302 | 0.0003 | 0.0003 | 0.9690 | 0.0003 | 3 | 476 | 0.0025 | 0.0004 | 0.9945 | 0.0014 | 0.0012 | 5 |
| 237 | 0.0026 | 0.0003 | 0.0004 | 0.9964 | 0.0003 | 3 | 477 | 0.0344 | 0.0006 | 0.9563 | 0.0041 | 0.0045 | 5 |
| 238 | 0.0019 | 0.0003 | 0.0003 | 0.9972 | 0.0003 | 3 | 478 | 0.0436 | 0.0006 | 0.9440 | 0.0044 | 0.0074 | 5 |
| 239 | 0.0074 | 0.0006 | 0.1532 | 0.8384 | 0.0004 | 3 | 479 | 0.0033 | 0.0004 | 0.9942 | 0.0011 | 0.0009 | 5 |
| 240 | 0.0045 | 0.0003 | 0.1532 | 0.8416 | 0.0004 | 3 | 480 | 0.1396 | 0.0006 | 0.8567 | 0.0013 | 0.0018 | 5 |
