# Supplementary material for: EST-SSR Primer Development and Genetic Structure Analysis of Psathyrostachys juncea Nevski
Source: Front Plant Sci. 2022 Feb 28;13:837787. doi: 10.3389/fpls.2022.837787 (PMC8919075; doi:10.3389/fpls.2022.837787)
Supplement: Supplementary file 6 [file Table_6.DOCX]

| Group | *F_st_* | Expected heterozygosity |
| --- | --- | --- |
| 1 | 0.4225 | 0.2495 |
| 2 | 0.4442 | 0.2310 |
| 3 | 0.4587 | 0.2036 |
| 4 | 0.5487 | 0.1721 |
| 5 | 0.3370 | 0.2701 |

# **Supplementary Table 6.** Expected heterozygosity and genetic differentiation coefficient (*F_st_*) in the population structure

Note: *F_st_* : population genetic differentiation coefficient; Expected heterozygosity: Average distances between individuals in same cluster.
