# Supplementary material for: EST-SSR Primer Development and Genetic Structure Analysis of Psathyrostachys juncea Nevski
Source: Front Plant Sci. 2022 Feb 28;13:837787. doi: 10.3389/fpls.2022.837787 (PMC8919075; doi:10.3389/fpls.2022.837787)
Supplement: Supplementary file 7 [file Table_7.DOCX]

# **Supplementary Table 7.** Simple statistics of measured values of various traits of *P. juncea*

|  | **Sample 1** | | | | **Sample 2** | | | |
| --- | --- | --- | --- | --- | --- | --- | --- | --- |
| **Trait** | **Mean** | **Minimum value** | **Maximum value** | **Coefficient of variation（%）** | **Mean** | **Minimum value** | **Maximum value** | **Coefficient of variation（%）** |
| Height (cm) | 23.27 | 13.25 | 47.18 | 25.71 | 20.77 | 11.93 | 30.50 | 21.62 |
| Basal cluster diameter (cm) | 18.84 | 11.50 | 25.50 | 15.77 | 14.44 | 8.83 | 22.63 | 26.64 |
| Nutritional tiller number | 52.00 | 29.00 | 91.00 | 33.10 | 26.00 | 12.00 | 46.00 | 38.13 |
| Tiller angle (°) | 36.92 | 25.00 | 55.00 | 22.07 | 41.21 | 16.25 | 61.25 | 20.75 |
| Leaf length (cm) | 21.49 | 13.95 | 28.93 | 15.87 | 21.68 | 16.11 | 31.15 | 14.41 |
| Leaf width (cm) | 0.43 | 0.31 | 0.53 | 11.87 | 0.44 | 0.32 | 0.53 | 10.46 |
| Canopy diameter (cm) | 35.30 | 20.75 | 51.18 | 20.71 | 36.13 | 21.00 | 50.00 | 22.98 |

Note: Tillering traits were measured on the materials of 60 individual plants in two samples. All traits were measured with five repetitions per plant.
